# Flexible prediction of opponent motion with internal representation in interception behavior

**DOI:** 10.1007/s00422-021-00891-9

**Published:** 2021-08-11

**Authors:** Kazushi Tsutsui, Keisuke Fujii, Kazutoshi Kudo, Kazuya Takeda

**Affiliations:** 1 Graduate School of Informatics, Nagoya University, Nagoya, Japan; 2 Graduate School of Arts and Sciences, The University of Tokyo, Tokyo, Japan; 3 RIKEN Center for Advanced Intelligence Project, Tokyo, Japan; 4 PRESTO, Japan Science and Technology Agency, Tokyo, Japan; 5 Graduate School of Interdisciplinary Information Studies, The University of Tokyo, Tokyo, Japan; 6 Institutes of Innovation for Future Society, Nagoya University, Nagoya, Japan

**Keywords:** Prediction, Extrapolation, Interception, Pursuit, Navigation, Internal representation

## Abstract

Skilled interception behavior often relies on accurate predictions of external objects because of a large delay in our sensorimotor systems. To deal with the sensorimotor delay, the brain predicts future states of the target based on the current state available, but it is still debated whether internal representations acquired from prior experience are used as well. Here we estimated the predictive manner by analyzing the response behavior of a pursuer to a sudden directional change of the evasive target, providing strong evidence that prediction of target motion by the pursuer was incompatible with a linear extrapolation based solely on the current state of the target. Moreover, using neural network models, we validated that nonlinear extrapolation as estimated was computationally feasible and useful even against unknown opponents. These results support the use of internal representations in predicting target motion, suggesting the usefulness and versatility of predicting external object motion through internal representations.

**Supplementary Information:**

The online version contains supplementary material available at 10.1007/s00422-021-00891-9.

## Introduction

Skilled motor behavior often relies on accurate predictions of external objects and environments (Wolpert and Flanagan 2001; Yarrow et al. 2009). For example, to hit a moving ball with a bat or capture an evasive opponent, it is essential to accurately predict target motion as well as own motion (Land and McLeod 2000; Brault et al. 2012; Fujii et al. 2014). In such cases, it would be easy to intercept a stationary or slow moving target, but may be difficult to intercept a fast moving target because there is a large delay in our sensorimotor systems. Sensorimotor delay, which is associated with receptor transduction, neural conduction, central processing and muscle activation, is inevitable in animals, and can be several hundreds of milliseconds in human interception behavior (Smeets and Brenner 1994; Franklin and Wolpert 2011). As a result, for the successful interception of fast moving targets, we would need to compensate for this sensorimotor delay by a prediction of future states (e.g., position and velocity) based on the current state available. The predictive mechanism to deal with sensorimotor delay is a prominent problem in interception behavior, and numerous studies have been done on predictions of target motion (Hayhoe 2017; Brenner and Smeets 2018; De la Malla et al. 2019; Fiehler et al. 2019). However, the understanding of target motion prediction is rather limited because these have predominantly studied target objects that move in a predictable manner, such as at a constant velocity (Brenner and Smeets 1996, 2007, 2009, 2015a, b; Brenner et al. 1998, 2013; Brouwer et al. 2000, 2002; De Lussanet et al. 2001) or accelerated by gravity (Lacquaniti and Maioli 1989; Zago et al. 2004, 2009, 2010; Senot et al. 2005, 2012; Zago and Lacquaniti 2005; López-Moliner et al. 2010; López-Moliner and Brenner 2016); it remains an open question how we predict interactive targets such as evasive opponents, which appear to be less predictable.

When we try to intercept an opponent, there are two basic manners that the brain may use to predict the future state of the opponent. One is linear extrapolation, and the other is nonlinear extrapolation. The first predictive manner has generally been assumed to estimate a target motion (or trajectory) based only on the current sensory information, namely position and velocity, of the target. That is, in this manner, the pursuer predicts that the target would move straight ahead regardless of the situation, in each instant. This linear extrapolation, which assumes a simple mechanism to compensate for the sensorimotor delay, is consistent with experimental observations in both humans (Engel et al. 1999; Engel and Soechting 2000) and non-humans (Borghuis and Leonardo 2015). The alternative manner has been assumed to estimate target motion based on internal representations in addition to the current information. That is, target motion is estimated through a mapping between the current state and the future state. Such transformations (or representations) are termed internal models and are thought to be acquired through prior experience. This manner allows us to make nonlinear extrapolation with our perceptible information and is supported by the fact that we can successfully catch a falling ball (Zago et al. 2004; López-Moliner and Brenner 2016). Specifically, even though the human visual system is poor at perceiving acceleration, the brain can accurately predict the motion of a ball accelerated by gravity. Although the use of internal models in predicting target motion is still controversial (Baurès et al. 2007; Zago et al. 2008), the idea is attractive in that it has the potential to predict target motion with greater accuracy by nonlinear extrapolation. However, it is unclear whether nonlinear extrapolation with an internal model is used for predicting the motion of opponents, who seem to have less stable rules of motion than free-falling objects, and, if so, whether it can accurately predict the opponent motion.

To address these questions, we conducted an experiment in which participants played a one-on-one chase and escape task on a screen with joystick controllers. Three experimental conditions for the width of the pitch (narrow, square, and wide) were studied to examine the situational dependence of the predictive manners. We analyzed the response behavior of the pursuer (defender) to a sudden directional change of the target (attacker) to estimate the predictive manner adopted by the pursuer, providing strong evidence that the pursuer would make a nonlinear extrapolation of the opponent motion. Moreover, we validated the feasibility and effectiveness of nonlinear extrapolations using neural network models which learn the mapping between the current state and the future state from the experimental data. Our results suggest the usefulness and versatility of the prediction of external objects through internal representations, and provide an insight into the predictability of others' behavior.

## Methods

### Participants

Twelve males participated in the experiment (aged 22–31, mean = 25.9, s.d. = 3.0). All participants were right-handed, had normal or corrected-to-normal vision, had some experience in amateur sports, and were naïve to the purpose of the study. This study was approved by the Ethics Committee of the University of Tokyo of Arts and Sciences. Informed consent was obtained from each participant before the experiments. Participants were recruited in pairs and every member of each pair took in turn the roles of both attacker and defender. They each received 1,000 yen per hour as a reward.

### Apparatus and stimuli

Participants were seated in a chair, and they operated the joystick of an Xbox One controller that could tilt freely in any direction to control a disk on the screen. The stimuli were presented on a 27-inch monitor (ASUS SWIFT PG278Q) at a refresh rate of 120 Hz. A black rectangle surrounding the disks was defined as the play area, or “pitch.” The width of the pitch was 7.5, 15.0, 30.0 cm in the narrow, square, and wide condition, respectively, with a consistent height of 15.0 cm. The velocity of each disk on the screen was determined by the degree of inclination of the joystick on their respective controllers. The maximum speed of both the attacker and the defender was set to 5.5 cm per second. The diameter of each disk was 1.0 cm, and the central position of each disk on the screen during the trials was recorded at 120 Hz on a computer (MacBook Pro) with Psychtoolbox version 3.0. The viewing distance of the participants was about 50 cm, and a partition prevented direct viewing of the hands or controller of the other player.

### Procedure and design

**Fig. 1:**
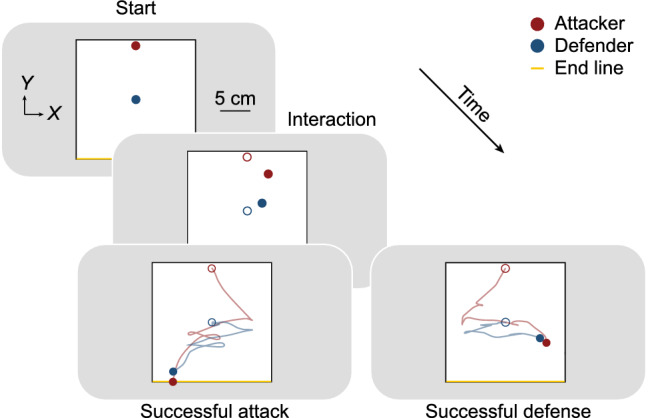
Experimental setup. Illustration of the experimental task. Participants (*n* = 12) controlled either an attacker (red disk) or a defender (blue disk) on a screen using the joystick of a controller. The initial location of the attacker was the upper middle (red circle) and that of the defender was the middle (blue circle) of the pitch (upper panel). The participant controlling the attacker was asked to move past the defender and reach the end line (lower left panel), whereas the participant controlling the defender was asked to “catch” (contact) the attacker without him reaching the end line (lower right panel). If the attacker moved out of pitch boundaries (black rectangle), the trial was deemed a successful defense

Each participant controlled either a red disk representing an attacker or a blue disk representing a defender on the screen (Fig. 1). The participant controlling the attacker was asked to get past the defender and reach the end line (yellow line) behind the defender (Fig. 1, lower left panel), whereas the participant controlling the defender was asked to catch the attacker without him reaching the end line (Fig. 1, lower right panel). "Catch" was defined as contact between the outer disk edges. If the attacker left the boundaries of the pitch (black rectangle), the trial was deemed a successful defense. The start trial position of the attacker was in the upper middle of the pitch (red circle), while that of the defender was in the center of the pitch (blue circle). The experimental task began with a start cue. A high-pitched beep sounded as feedback to a successful attack. Conversely, a low-pitched beep sounded after a successful defense. The number of successful attacks was indicated at the end of each block. The experimental block consisted of 50 trials, with a warm-up of 10 trials to get used to the task. There were three experimental conditions (narrow, square, and wide), and each participant played one block in turn on both the attacker and the defender under each experimental condition. In total, there were 60 warm-up trials and 300 experimental trials for each participant (or each pair). The order of the experimental conditions was counterbalanced across pairs.

### Behavioral analysis

**Fig. 2:**
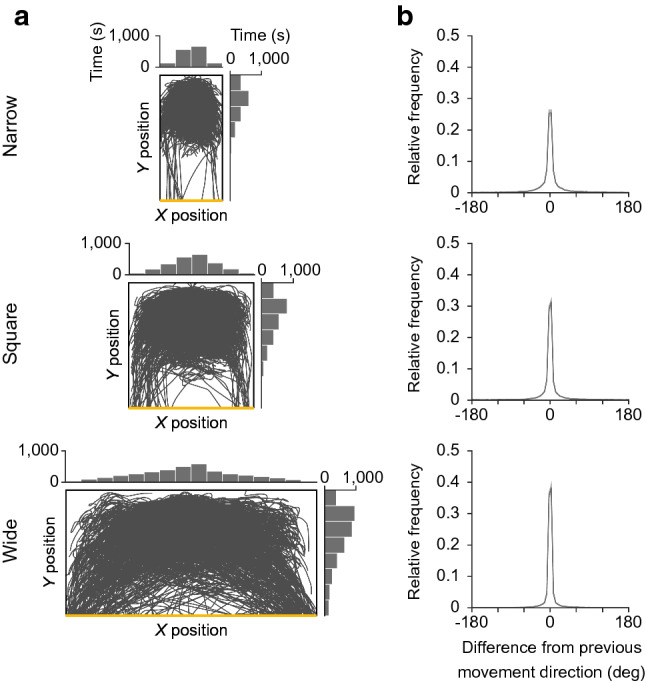
Characteristics of target motion. **a** Attacker paths with histograms of *X* and *Y* positions, in all trials (*n* = 600) for each of the experimental conditions (narrow, square, and wide). Bin width was set to divide the pitch into 4, 8, and 16 columns for the three conditions, respectively. **b** Relative frequency distribution of the difference in attacker moving direction between at time *t* and *t* + 1 (∆*t* = 50 ms) for each of the three experimental conditions. Bin width was set to 5 degrees. To quantify the unpredictability of attacker motion, we calculated the entropy. One-way repeated-measures ANOVA with the Holm–Bonferroni method was used (*F*_(2, 22)_ = 91.152, *P* < 0.001, *η*^2^ = 0.800; narrow vs. square: *t*_11_ = 5.835, *P* < 0.001; square versus wide: *t*_11_ = 8.745, *P* < 0.001; narrow versus wide: *t*_11_ = 11.533, *P* < 0.001)

We recorded the onscreen *X* and *Y* positions of the attacker and defender. All behavioral analyses, except for the evaluation of unpredictability of target motion (Fig. 2b), were performed using data recorded at 120 Hz. Because we thought it would be reasonable to use data with a temporal resolution closer to human perception in evaluating the unpredictability of the target motion, we used the downsampled data in this analysis. Specifically, we first downsampled the recorded data to 20 Hz based on the previous studies (Pöppel 1997; Mrotek and Soechting 2007a, b) and then, using the downsampled data, we calculated the difference in the target (attacker) moving direction between time $t$ and time $t + 1$ ($\Delta t = 50$ ms) and the entropy (see Supplementary Fig. 1). The following behavioral analyses are performed using data recorded at 120 Hz. Directional change was defined as velocity in the *X* direction crossing zero, and response time as the temporal difference between the directional changes of the attacker and the defender, distinguishing between positive and negative *X* velocities. The response time might be affected by the movement directions of both attacker and defender, but since our interest was in the approximate value, we only focused on the time difference for simplicity. We limited the range of response times from 0 to 500 ms, and removed any response times longer than 500 ms from the analyses to exclude responses where the defender had given up trying to catch the attacker. A short latency response was defined as the response less than 150 ms based on the results of the simple reaction task (see Supplementary Figs. 2 and 3). To calculate the values of each variable within each horizontal position on the pitch, we divided the pitch into 4, 8 and 16 columns for the three experimental conditions, respectively. For the column that containing missing values, such as because the participants did not go to that position, the mean and s.e.m. were calculated excluding the missing values, which are shown by dotted circles and lines in each figure. The frequency of directional change was defined as the average number of occurrences per second. Specifically, the frequency was calculated by dividing the number of directional changes by the time spent in each *X* column. Note that we focused mainly on the direction of movement in the behavioral analysis, because both attackers and defenders were moving at almost maximum speed most of the time; the proportion of movement speed that exceeded 90% of the maximum speed was more than 90% in all conditions for both attackers and defenders.

### Computational model

Because we thought it would be reasonable to use data with a temporal resolution closer to human perception in modeling human prediction, we also used the data downsampled to 20 Hz in the analyses of the computational model. At every time *t*, the neural network models receives an input vector ${\varvec{x}}_{t}$ and emits a hidden state vector from the last layer ${\varvec{h}}_{t}^{l}$ that parameterizes a predictive distribution of the target position $\hat{\user2{y}}_{t}$ at next time-step $t + 1$ ($\Delta t = 50$ ms) relative to the current position. To correspond to the available sensory information of the pursuer (or defender) as assumed in previous research, the input vector ${\varvec{x}}_{t} \in {\mathbb{R}}^{6}$ in this study is composed of position and velocity information. Specifically, this consisted of the velocity vectors of the attacker ${\varvec{v}}_{t}^{a} = \left( {v_{X}^{a} , v_{Y}^{a} } \right)_{t}$ and defender ${\varvec{v}}_{t}^{d} = \left( {v_{X}^{d} , v_{Y}^{d} } \right)_{t}$ and a range vector ${\varvec{r}}_{t} = \left( {r_{X} , r_{Y} } \right)_{t}$, which is defined as a vector pointing from the position of the pursuer to that of the target. Our three neural network models contained three weight layers. The dimensions of the hidden state vectors of the first layer ${\varvec{h}}_{t}^{f} \in {\mathbb{R}}^{64}$, second layer ${\varvec{h}}_{t}^{s} \in {\mathbb{R}}^{128}$, and last layer ${\varvec{h}}_{t}^{l} \in {\mathbb{R}}^{5}$ were determined according to previous research (Alahi et al. 2016). The hidden state vector of the last layer ${\varvec{h}}_{t}^{l}$ was used to generate a bivariate Gaussian distribution parameterized by the mean ${\varvec{\mu}}_{t} = \left( {\mu_{X} , \mu_{Y} } \right)_{t}$, standard deviation ${\varvec{\sigma}}_{t} = \left( {\sigma_{X} , \sigma_{Y} } \right)_{t}$, and correlation coefficient $\rho_{t}$ following the previous researches (Graves 2013; Alahi et al. 2016). The predicted distribution $\hat{\user2{y}}_{t}$ at time *t* is given by $\hat{\user2{y}}_{t} \sim {\mathcal{N}}\left( {{\varvec{\mu}}_{t} , {\varvec{\sigma}}_{t} , \rho_{t} } \right)$.

In our neural network models, the input and hidden layers differed among the networks, while the output layer was common. In the LN model, all layers were composed of the fully connected layer without nonlinearity,

$$ {\varvec{h}}_{t} = {\varvec{W}}_{xh} {\varvec{x}}_{t} + {\varvec{b}}_{h} $$

where ${\varvec{W}}_{xh}$, and ${\varvec{b}}_{h}$ denote the input-to-hidden weight matrix and the bias vector, respectively. In the NN model, only the output layer is the fully connected layer without nonlinearity, and the other layers are composed of the fully connected layers with nonlinearity,

$$ {\varvec{h}}_{t} = \varphi \left( {{\varvec{W}}_{xh} {\varvec{x}}_{t} + {\varvec{b}}_{h} } \right) $$

where $\varphi \left( x \right) = {\text{max}}\left( {0,x} \right)$ is the rectified linear unit (ReLU) for nonlinearity (Glorot et al. 2011). In the recurrent neural network (RNN) model, the input, output, and hidden layers are, respectively, the fully connected layer without nonlinearity, that with nonlinearity, and a recurrently connected layer,

$$ {\varvec{h}}_{t} = {\text{tanh}}\left( {{\varvec{W}}_{xh} {\varvec{x}}_{t} + {\varvec{W}}_{hh} {\varvec{h}}_{t - 1} + {\varvec{b}}_{h} } \right) $$

where ${\varvec{W}}_{hh}$ is the hidden-to-hidden (or recurrent) weight matrix and ${\varvec{h}}_{t - 1}$ is the hidden state vector at the previous time-step $t - 1$.

The LSTM model was designed to be better at storing and accessing information than standard RNNs, and the hidden layer of the RNN model is replaced with an LSTM layer below,

$$ \begin{aligned} {\varvec{i}}_{t} & = \sigma \left( {{\varvec{W}}_{xi} {\varvec{x}}_{t} + {\varvec{W}}_{hi} {\varvec{h}}_{t - 1} + {\varvec{b}}_{i} } \right) \\ {\varvec{f}}_{t} & = \sigma \left( {{\varvec{W}}_{xf} {\varvec{x}}_{t} + {\varvec{W}}_{hf} {\varvec{h}}_{t - 1} + {\varvec{b}}_{f} } \right) \\ {\varvec{o}}_{t} & = \sigma \left( {{\varvec{W}}_{xo} {\varvec{x}}_{t} + {\varvec{W}}_{ho} {\varvec{h}}_{t - 1} + {\varvec{b}}_{o} } \right) \\ {\varvec{c}}_{t} & = {\varvec{f}}_{t} \odot {\varvec{c}}_{t - 1} + {\varvec{i}}_{t} \odot \tanh \left( {{\varvec{W}}_{xc} {\varvec{x}}_{t} + {\varvec{W}}_{hc} {\varvec{h}}_{t - 1} + {\varvec{b}}_{c} } \right) \\ {\varvec{h}}_{t} & = {\varvec{o}}_{t} \odot {\text{tanh}}\left( {{\varvec{c}}_{t} } \right) \\ \end{aligned} $$

where $\sigma \left( x \right) = 1/\left( {1 + {\text{exp}}\left( { - x} \right)} \right)$ is the logistic sigmoid function, ***i***, ***f***, ***o***, ***c***, and ***h*** are the input gate, forget gate, output gate, memory cell, and hidden state activation vectors, respectively, at time-step *t*. ${\varvec{h}}_{0} = {\varvec{c}}_{0} = 0$. The ${\varvec{W}}$ terms denote weight matrices, the ${\varvec{b}}$ terms are biases, and ⊙ is the Hadamard (element-wise) product. The deep neural network models (DNN, DRNN, and DLSTM) had two hidden layers each.

The neural network models were trained to minimize the loss ${\mathcal{L}} = - \mathop \sum \nolimits_{t = 1}^{T} {\text{log }}{\mathbb{P}}\left( {{\varvec{y}}_{t} {|}{\mathcal{N}}\left( {{\varvec{\mu}}_{t} , {\varvec{\sigma}}_{t} , \rho_{t} } \right)} \right)$ where ${\varvec{y}}_{t}$ ($= {\varvec{x}}_{t + 1}$) denotes the actual target position at next time-step $t + 1$. Network parameters were iteratively optimized via stochastic gradient descent with the Adam optimizer (Kingma and Ba 2015). The learning rate and batch size was 0.0003 and 16, respectively, in all neural network models and experimental conditions. These hyper-parameters were selected using a grid search on pre-experimental data (Supplementary Table 1) to make full use of the experimental data. The networks were trained for the experimental data of 11 participants (550 trials) and tested on the experimental data of the other one participant (50 trials) in each model and condition; that is, model performance was evaluated by “leave-one participant-out cross-validation.” The average number of training data (time-steps) was 27,428 (range: 26,465–28,551), and that of the test data was 2493 (range: 1370–3456).

To evaluate model performance using the test data, ${\varvec{\mu}}_{t}$ was used for the predicted coordinates $\hat{\user2{y}}_{t}$ in the one time-step prediction and compared with the actual coordinates ${\varvec{y}}_{t}$. The predicted coordinates $\hat{\user2{y}}_{t}$, and the displacement to reach them, were used as model input for position and velocity of the attacker at the next time-step $t + 1$ in the sequential (or trajectory) prediction. In this case, we assumed that own state (position and velocity of the defender) could be used up to 250 ms ahead based on estimations with the internal model of own motion (Wolpert et al. 1998, 2011; Kawato 1999; Imamizu et al. 2000). In addition, in the sequential prediction, for RNN, LSTM, DRNN, and DLSTM models, a 2.5 s observational period was provided to “warm” the hidden state. The prediction and the observation for it were kept within the same trial, and never crossed between trials.

The linear (L) model predicts that the target continued to move in the same direction at a constant speed. We first calculated the target displacement from time $$ t - 1$$ to the current time $$t$$, and added the displacement to the current position to predict the position at time $$t + 1$$. The curvilinear (C) model predicts that the target continues to move at the same speed and angular velocity along a circular arc. We thus calculated the target displacements from time $$t - 2$$ to time $$t - 1$$ and that from time $$t - 1$$ to the current time $$t$$, and then computed the angular change $$\Delta \theta$$ per a time-step using the displacements. When predicting target position at time $$t + 1$$, movement speed is equal to the magnitude of the latest displacement and movement direction is the angle of the latest displacement plus $$\Delta \theta$$. Consequently, for $$\Delta \theta = 0$$, the predictions of the linear and curvilinear models are equal.

### Statistical analysis

**Fig. 3:**
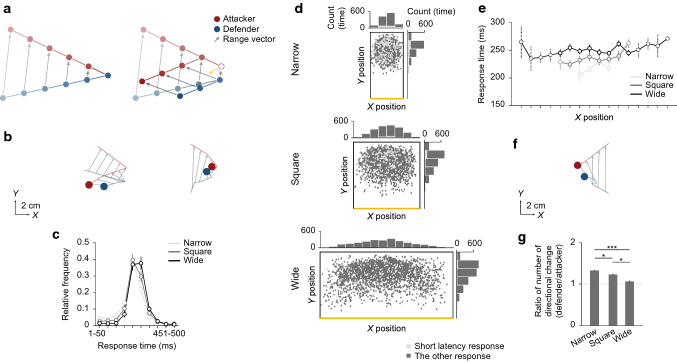
Anticipatory response to directional change of target movement by pursuer. **a** Traditional description of pursuit of a target that moves straight (left panel), and a target that changes movement direction (right panel). The pursuer (blue disk) often moves along the time-optimal (shortest) path to intercept a target (red disk). The gray arrow denotes the range vector from pursuer to target at each instant. Assuming that the pursuer predicts the near-future position of the target using a linear extrapolation (red circle), the directional change of the pursuer is necessarily delayed by one step from that of the target due to sensorimotor delay of the pursuer. **b** Example trajectories of passive (left panel) and anticipatory (right panel) responses. In many cases, the pursuer reactively changed its moving direction with respect to that of the target, while the pursuer, in some case, changed its moving direction anticipatory. **c** Relative frequency distribution of the response times. Response time was defined as the temporal difference between the target and pursuer in the zero-crossing of their horizontal velocities. Bin width was set to 50 ms. We compared the proportion of the short latency response (less than 150 ms). One-way repeated-measures ANOVA with the Holm–Bonferroni method was used (*F*_(2, 22)_ = 17.386, *P* < 0.001, *η*^2^ = 0.427; narrow versus square: *t*_11_ = 3.012, *P* = 0.024; square versus wide: *t*_11_ = 3.014, *P* = 0.024; narrow versus wide: *t*_11_ = 5.685, *P* < 0.001). **d** Spatial distribution and histogram of response for each of the experimental conditions. Light and dark gray dots denote the short and other latency responses, respectively. Bin width was set to divide the pitch (horizontal position) into 4, 8 and 16 columns for the respective experimental conditions. **e** Mean response times within each horizontal position on the pitch. Bin width was set to divide the pitch into 4, 8, and 16 columns for the respective experimental conditions. Dashed circles and error bars denote bins containing missing values and that were excluded from statistical analysis. Because the number of bins was different across the conditions and we were interested in differences of response times within the pitch, we used one-way repeated-measures ANOVA with the Holm–Bonferroni method for each experimental condition (*F*_narrow(3, 33)_ = 1.958, *P* = 0.140, *η*^2^ = 0.075; *F*_square(2.16, 23.72)_ = 1.064, *P* = 0.365, *η*^2^ = 0.033; *F*_wide(3.72, 40.95)_ = 2.715, *P* = 0.046, *η*^2^ = 0.076). For detailed statistics, see Supplementary Table 2. **f** Example trajectory of anticipatory response failure. In this case, the pursuer probably changed its moving direction in an incorrect anticipation of a directional change by the target. **g** Ratio of directional changes in the horizontal (*X*) position of the defender versus that of the attacker. One-way repeated-measures ANOVA with the Holm–Bonferroni method was used (*F*_(2, 22)_ = 66.279, *P* < 0.001, *η*^2^ = 0.738; narrow vs. square: *t*_11_ = 4.124, *P* = 0.017; square vs. wide: *t*_11_ = 8.545, *P* < 0.001; narrow vs. wide: *t*_11_ = 9.966, *P* < 0.001). For all panels, quantitative data represent the mean ± s.e.m across participants. *P < 0.05; ***P < 0.001

No statistical methods were used to predetermine sample sizes, but our sample sizes were chosen based on standards in the field. All quantitative data are reported as mean ± s.e.m. across participants. The data were analyzed using one-, two- or three-way repeated-measures analysis of variance (ANOVA), as appropriate. For these tests, Mauchly’s test was used to test sphericity; if the sphericity assumption was violated, degrees of freedom were adjusted by the Greenhouse–Geisser correction. *P* values were adjusted by the Holm–Bonferroni method for multiple comparisons. The column containing missing values was excluded from statistical analyses (Figs. 3e, 6a). The data distribution was assumed to be normal for multiple comparisons, but this was not formally tested. Two-tailed statistical tests were used for all applicable analyses. The significance level was set at an alpha value of 0.05. The method of Holm was used to adjust the *P* values in multiple testing (Holm 1979). When reporting *K P* values for *K* distinct tests, the Holm method is to compare the *r*th smallest *P* value (for *r* = 1,...,*K*) among the *K P* values with 0.05/(*K* − *r* + 1), and the test result is considered statistically significant after adjustment for the multiple tests if the *r*th smallest *P* value is less than 0.05/(*K* − *r* + 1). However, if the *r*th smallest *P* value is the first that exceeds 0.05/(*K* − *r* + 1), then the test results associated with the (*K* − *r* + 1) largest *P* values are considered statistically nonsignificant according to the Holm method. To make the presentation simpler, we let the adjusted *P* value be (*K* − *r* + 1) times the original *P* value and simply compare the adjusted *P* value with 0.05 to determine whether a particular test result is statistically significant after adjustment. Specific test statistics, *P* values, and effect sizes for the analyses are detailed in the corresponding figure legends and in Supplementary Table 2. All statistical analyses were performed using R version 4.0.2 (The R Foundation for Statistical Computing).

## Results

Our task required participants to control either an attacker (target; red disk) or a defender (pursuer; blue disk) on a screen using the joystick of a controller (Fig. 1). The participant controlling the attacker was asked to move past the defender and reach the end line (Fig. 1, lower left panel). On the other hand, the participant controlling the defender was asked to catch the attacker before the attacker reached the end line. A "catch" was regarded as a case where the outer edges of the disks were in contact (Fig. 1, lower right panel). If the attacker moved out of the pitch bounds (black rectangle), the trial was deemed a successful defense. The velocity of each disk on screen was determined by the degree of joystick inclination on the respective controllers, and the disks had equal maximum speed (magnitude of velocity). Three experimental conditions (narrow, square, and wide) were set to examine whether the predictive manner changed for targets with different rules of motion; based on the previous research (Tsutsui et al. 2019a), we reasoned that targets would change direction more frequently in the narrow pitch condition, whereas move more linearly in the wide pitch condition. The proportion of successful defenses were 0.97, 0.91, and 0.59, respectively, with mean trial durations of 2.47, 3.99, and 6.38 s, for each experimental condition.

### Characteristics of target motion

We first examined the characteristics of the target motion. The target (attacker) paths show highly varied motion (Fig. 2a). To quantify the unpredictability of target motion (or the effectiveness of a linear extrapolation) from the perspective of the pursuer (defender), we calculated the difference in movement direction of the target between at a time $t$ and time $t + 1$ ($\Delta t = 50$ ms) for each experimental condition and calculated the entropy (Fig. 2b). As expected, the proportion of linear movement of the target decreased as the pitch narrowed. This indicates that linear extrapolation would not work effectively as the pitch narrowed.

### Anticipatory response to target movement by pursuer

Then, to determine the predictive manner used by the pursuer to extrapolate the target motion, we analyzed the response behavior of the pursuer to sudden directional changes of the target. Pursuit behaviors, from insects to mammals, have often been described as movements toward the estimated future position of the target based on its current position and velocity (Olberg et al. 2000; Fajen and Warren 2004; Ghose et al. 2006; Olberg 2012; Kane et al. 2015; Tsutsui et al. 2019b) (Fig. 3a, left panel). In other words, this description (or model) assumes a linear extrapolation of target motion by pursuers in each moment. Accordingly, the directional change of the pursuer would necessarily be one step behind that of the target owing to the sensorimotor delay (Fig. 3a, right panel). Conversely, the temporal difference of directional changes between the target and pursuer allows us to estimate the predictive manner of the pursuer. Specifically, if a pursuer adopts linear extrapolation to estimate the future target position (or motion), the response of the pursuer to a directional change of the target should be purely reactive, whereas this would not necessarily be the case when adopting nonlinear extrapolation, as the response may include anticipatory components (e.g., extremely short latency response).

Thus, we examined the temporal differences in directional changes in the horizontal (*X*) position between the target and pursuer. The directional change of the pursuer was basically reactive, occurring after perceiving that of the target (Fig. 3b, left panel), but, in some case, was anticipatory, occurring before perceiving that of the target (Fig. 3b, right panel). The frequency distribution of the response times, defined as the temporal difference between the target and pursuer in the zero-crossing of their horizontal velocities, included extremely short latency responses (Fig. 3c), and the proportion of short latency responses (less than 150 ms) increased as the pitch width narrowed (0.14, 0.09, and 0.04, respectively). These results strongly indicate that a linear extrapolation of target motion was insufficient to explain the predictive manner of the pursuer. It also indicates that the pursuers may flexibly change their predictions depending on the target motion or situation.

To examine the spatial factors that result in a short latency response, we next focused on its spatial distribution. In studies on eye movement, explicit barriers are known to promote anticipatory eye movements (Kowler 1989; Kowler et al. 2014, 2019). If the pursuer’s short latency responses would be distributed near the edges of the pitch, an explicit barrier may have been employed. However, the short latency responses were distributed at the middle as well as the edges of the pitch (Fig. 3d), and the mean response times for the horizontal position on the pitch were almost uniform (Fig. 3e). These results indicate that the defender made a short latency response even in situations where the information on explicit barriers would be difficult to use for prediction, suggesting that a short latency response, that is, a nonlinear extrapolation of target motion, may involve using clues from rules of target motion rather than explicit barriers. In addition, we found the cases in which the directional change of the pursuer failed in anticipation of that of the target (Fig. 3f). The ratio of the directional changes of the pursuer versus the target was greater than 1 for each experimental condition (Fig. 3g). If the defender uses a linear extrapolation (i.e., behave passively), the ratio of the directional change of defender to that of attacker should be equal to (or less than) 1. On the other hand, if the defender makes an incorrect nonlinear extrapolation (as shown in Fig. 3f), the ratio could exceed 1. This result therefore suggests that anticipatory responses with nonlinear extrapolations were attempted under all experimental conditions, but that such nonlinear extrapolations were not always spatiotemporally appropriate.

### Verification of predictability of target motion with neural network models

**Fig. 4:**
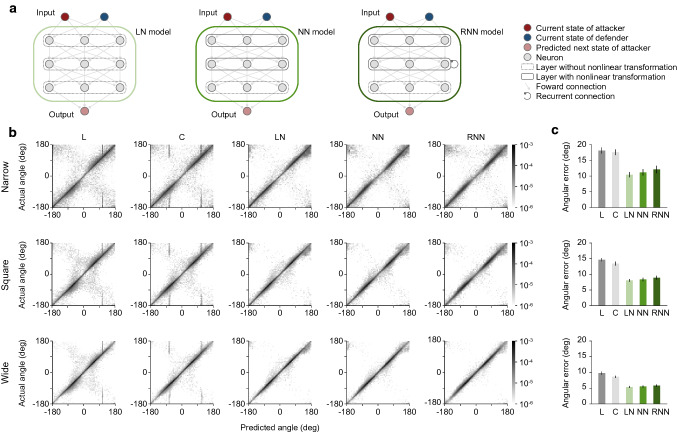
Prediction of target movement direction at the next time-step with models. **a** Illustration of neural network models. The models predicted a next state of attacker (target) using the current states of attacker and defender (pursuer). The linear neural network (LN) model was composed only of linear transformations (left panel). The nonlinear neural network (NN) model included nonlinear transformations (middle panel). The recurrent neural network (RNN) model had a recurrent structure in addition to the nonlinear transformation (right panel). **b** Two-dimensional relative frequency distribution between predicted and actual directions of target movement for each of the experimental conditions (Pooled data for all participants). L, C, LN, NN, and RNN denote the linear, curvilinear, linear neural network, nonlinear neural network, and recurrent neural network model, respectively. Bin width was set to 5 degrees. **c** Angular error of the model prediction for each of the experimental conditions. Two-way repeated-measures ANOVA with the Holm–Bonferroni method was used (*F*_condition(1.29, 14.18)_ = 68.184, *P* < 0.001, *η*^2^ = 0.393; *F*_model(1.60, 17.63)_ = 151.485, *P* < 0.001, *η*^2^ = 0.341; *F*_condition×model(1.91, 21.02)_ = 10.900, *P* < 0.001, *η*^2^ = 0.020). For detailed statistics, see Supplementary Table 2. For all panels, quantitative data represent the mean ± s.e.m across participants

We thus sought to determine whether short latency responses were coincidental by examining the predictability of target motion with neural network models which predict the future position of the target through a mapping between the current state and the future state in a learning-based method (Fig. 4a). The input of the neural network models corresponded to the sensory information often used in chase (Ghose et al. 2006; Kane et al. 2015) or escape (Domenici 2002) models, namely the velocity vectors of pursuer and target, and the range vector, which is defined as a vector pointing from the position of the pursuer to that of the target. The output was the estimated position of the target, as represented by a bivariate Gaussian distribution. Note that inputs of the neural network models did not include accelerations or information on explicit barriers such as distance to the edge of the pitch. The models were trained using the error-based algorithm, a comparison between the predicted and actual consequences of the target position, using data from all but one participant, whose data were used to test the fidelity of the model prediction. To obtain insights into the important information for accurate prediction of the target motion, we computed three neural network models. The linear neural network (LN) model was composed only of linear transformations, while the nonlinear neural network (NN) model included a widely-used nonlinear transformation (Glorot et al. 2011; Lecun et al. 2015). The recurrent (nonlinear) neural network (RNN) model had a recurrent structure in addition to the nonlinear transformation. Thus, if nonlinearity is necessary for accurate prediction, the performance of the NN model should outperform that of the LN model, and if recurrence (time-series information) is necessary for accurate prediction, the performance of the RNN model should outperform that of the NN model. For comparison, we also computed two extrapolation models, linear (L) and curvilinear (C), as proposed in previous studies (Mrotek and Soechting 2007a, b; Borghuis and Leonardo 2015).

We first examined the model accuracy in predicting the movement direction of the target at the next time-step. To visualize the association between the predicted and actual angles, we showed the two-dimensional relative frequency distribution (Fig. 4b). The angles in this figure represent the movement direction of the target with respect to the pursuer. This relative movement direction was defined between − 180 and 180 degrees, with 0 degree indicating movement directly toward the pursuer, and positive and negative values indicating movement to the left and right sides, respectively, with respect to the pursuer. As shown in this figure, the predicted and actual angles were roughly matched in all models, while some deviation was found especially where the angles had different signs (Fig. 4b, second and fourth quadrants). Note that the deviations were lower in the neural network models (LN, NN, and RNN) than in the conventional ones (L and C). On average, the neural network models showed better agreement between the angles than the conventional ones under all conditions (Fig. 4c). These results suggest that the neural network models can more accurately predict the target motion for various situations including sudden directional change (e.g., left to right, or vice versa) (see also Supplementary Fig. 4).

### Flexible and accurate longer-term prediction by neural network models

**Fig. 5:**
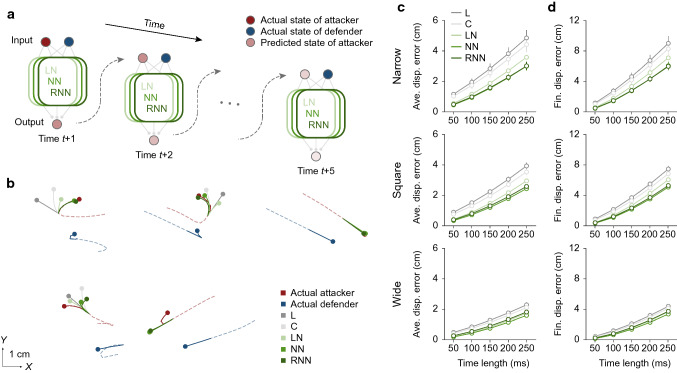
Sequential prediction of target motion with models. **a** Illustration of sequential prediction by neural network models. The predicted state of the attacker (target) was used as a part of model input at the next time-step, and the prediction was made sequentially up to 5 steps (250 ms) ahead. Assuming that the own state of the defender (pursuer) could be accurately estimated by the internal model of own motion, we used the actual state of the defender as a part of the model input for sequential prediction. The same procedure was used for all neural network models. **b** Examples of predicted and actual trajectories. L, C, LN, NN, and RNN denote the linear, curvilinear, linear neural network, nonlinear neural network, and recurrent nonlinear neural network models, respectively. Red and blue lines show the actual trajectory of attacker and defender, respectively. The disks denote the end points of the predicted and actual trajectories. For ease of visibility, the trajectories of attacker and defender from the 10 time-steps (500 ms) before, to the time of prediction start, are shown by dashed red and blue lines. **c** Average displacement error of the sequential model prediction up to 5 time-steps (250 ms) ahead for each of the experimental conditions. Three-way repeated-measures ANOVA with the Holm–Bonferroni method was used (*F*_condition(1.11, 12.26)_ = 21.006, *P* < 0.001, *η*^2^ = 0.144; *F*_model(1.18, 12.93)_ = 76.106, *P* < 0.001, *η*^2^ = 0.0952; *F*_time length(1.00, 11.04)_ = 471.038, *P* < 0.001, *η*^2^ = 0.511; *F*_condition×model(1.30, 14.29)_ = 8.486, *P* = 0.008, *η*^2^ = 0.015; *F*_model×time length(1.39, 15.33)_ = 44.454, *P* < 0.001, *η*^2^ = 0.009; *F*_condition×time length(1.12, 12.35)_ = 20.470, *P* < 0.001, *η*^2^ = 0.032; *F*_condition×model×time length(1.48, 16.25)_ = 6.433, *P* = 0.014, *η*^2^ = 0.002). For detailed statistics, see Supplementary Table 2. (**d**) Final displacement error of the sequential model prediction up to 5 time-steps (250 ms) ahead. Three-way repeated-measures ANOVA with the Holm–Bonferroni method was used (*F*_condition(1.12, 12.28)_ = 20.660, *P* < 0.001, *η*^2^ = 0.105; *F*_model(1.24, 13.61)_ = 62.994, *P* < 0.0001, *η*^2^ = 0.049; *F*_time length(1.01, 11.07)_ = 505.531, *P* < 0.001, *η*^2^ = 0.637; *F*_condition×model(1.34, 14.70)_ = 7.689, *P* = 0.010, *η*^2^ = 0.008; *F*_model×time length(1.44, 15.87)_ = 38.561, *P* < 0.001, *η*^2^ = 0.009; *F*_condition×time length(1.13, 12.39)_ = 19.900, *P* < 0.001, *η*^2^ = 0.036; *F*_condition×model×time length(1.48, 16.33)_ = 5.915, *P* = 0.017, *η*^2^ = 0.002). For detailed statistics, see Supplementary Table 2. For all panels, quantitative data represent the mean ± s.e.m across participants

Given that the sensorimotor delay during the pursuit was about 250 ms (see Fig. 3c), it would be desirable to be able to predict 250 ms ahead to spatiotemporally match our own motion with the target motion accurately for successful interception. Thus, we then examined the model performance of sequential prediction for the target motion by testing the prediction accuracy up to 250 ms ahead in each model (Fig. 5a). In this analysis, the estimated target state (position and velocity) at time $t$ was used sequentially as input for the prediction at next time-step $t + 1$, up to 250 ms (5 time-steps) ahead. Representative examples show that the neural network models were able to accurately predict a variety of trajectories, including the straight, gentle curve, and sharp curve phases (Fig. 5b, upper panels). Even though the predictions were occasionally incorrect (Fig. 5b, lower panels), on average, the neural network models made more accurate predictions than the conventional ones for both the average and final displacement errors (Fig. 5c, d). These results indicate that the neural network models also worked well in predicting target motion over a longer period of time and therefore would be of practical usefulness.

### Ineffectiveness of the recurrent structure

**Fig. 6:**
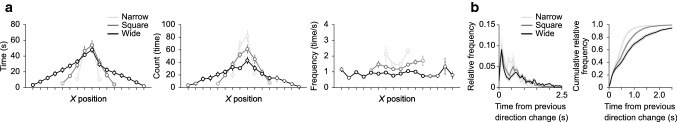
Spatiotemporal property of change in the horizontal direction of the target. (a) Frequency distribution of time spent of the attacker (left), frequency distribution of directional changes in horizontal position of the attacker (middle), and frequency distribution per time of directional changes (right) within each horizontal position on the pitch. Bin width was set to divide the pitch into 4, 8, and 16 columns for the respective conditions. Dashed circles and error bars denote that the bin contained missing values. Because the number of bins was different across the conditions and we were interested in differences of the frequency per time within the pitch, we used one-way repeated-measures ANOVA with Holm–Bonferroni method in each experimental condition (*F*_narrow(1.32, 14.52)_ = 2.870, *P* = 0.104, *η*^2^ = 0.150; *F*_square(2.16, 23.78)_ = 2.115, *P* = 0.140, *η*^2^ = 0.142; *F*_wide(3.66, 40.25)_ = 2.159, *P* = 0.096, *η*^2^ = 0.125). **b** Frequency distribution (left) and cumulative frequency distribution (right) of time from previous directional change. For all panels, quantitative data represent the mean ± s.e.m across participants

Although the RNN model appeared to contain richer information due to its recurrent structure, its predictive performance was similar to or slightly lower than that of the NN model. While we also examined whether predictive performance improved using the long short-term memory (LSTM) model, which can hold information for longer time periods, the prediction accuracy was almost the same (Supplementary Figs. 5, 6, and 7). To clarify why the recurrent structure did not lead to a performance improvement in the sequential prediction, we investigated the properties of target motion in terms of directional changes in the horizontal position. Based on the findings of the previous research (Tsutsui et al. 2019a), we focused on frequency distributions in spatial and temporal aspects regarding the change in the horizontal direction of the target. First, we investigated the spatial bias in the frequency per time of the directional change of the target and found that it was almost uniform within the pitch under all experimental conditions (Fig. 6a). Next, we investigated the relative frequency distribution of the time interval between directional changes and found that it decayed exponentially over time, particularly after a second peak at approximately 500 ms (Fig. 6b). These results indicate that directional changes in the horizontal position of the target showed the Poisson-like property known as “memorylessness” (see also Supplementary Fig. 8). In other words, whether the target changes movement direction in any moment may be little influenced by the prior process, and it suggests that this spatiotemporal property of target motion may be a reason why the recurrent structure did not lead to improvement of performance in the sequential prediction.

## Discussion

Traditionally, pursuit behavior, from insects to mammals, has been described as movement along a local shortest path toward the estimated future position of the target based on its current position and velocity (Land and Collett 1974; Olberg et al. 2000; Fajen and Warren 2004; Ghose et al. 2006; Olberg 2012; Kane et al. 2015; Tsutsui et al. 2019b). In these studies, it is often (implicitly) assumed that the motion (or trajectory) of a target is predicted by linear extrapolation, and under such an assumption, the pursuer (or defender) should be purely reactive to a sudden directional change of the target (or attacker). Here we have shown that, in striking contrast to these traditional descriptions, pursuers sometimes change their movement direction before perceiving (or even without occurring) a directional change of the target. Our results are consistent with a recent finding that pursuit behavior relies on predictions through target models (Mischiati et al. 2014), presenting the possibility that the predictive mechanisms that humans (or animals) use to compensate for sensorimotor delays during pursuit are more sophisticated than previously thought.

Previous studies on human interception behavior have predominantly used target objects moving in a predictable manner—at a constant velocity (Brenner and Smeets 1996, 2007, 2009, 2015a, b; Brenner et al. 1998, 2013; Brouwer et al. 2000, 2002; De Lussanet et al. 2001) or accelerated by gravity (Lacquaniti and Maioli 1989; Zago et al. 2004, 2009, 2010; Senot et al. 2005, 2012; Zago and Lacquaniti 2005; López-Moliner et al. 2010; López-Moliner and Brenner 2016). Presumably, the reason that these experimental paradigms have dominated, despite the fact that we often encounter less predictable situations in daily life or sports, is that these paradigms allow investigation under strict experimental controls. However, due to their simplicity, these paradigms may occasionally allow multiple interpretations for experimental observations. For example, in catching a falling ball, some researchers have proposed that an internal model, which allows us to extrapolate a nonlinear trajectory, is used to predict the target motion because we can catch a ball accelerated by gravity in spite of being poor at perceiving accelerations, while some others have questioned this proposal (Baurès et al. 2007). The question results from considerations that the capture of a falling ball can also be performed by continuous prediction using a linear extrapolation in each instance. Indeed, in this case, the predictions of the two predictive manners are not much different (Baurès et al. 2007). However, our experiment allowed us to distinguish between the two manners. Our results that pursuers anticipatorily changed their movement directions obviously cannot be explained by continuous linear extrapolation, and support the idea that an internal model is used to predict target motion.

Predictions of target motion through internal representations have an ability to comprehensively describe the experimental observations in various situations. We found that pursuers flexibly change their frequency of anticipating directional change of the target in response to the expectation that the target would go straight in each instance. This result implies that in situations where the target is always straight ahead, the pursuer will predict that the target moves in a straight line. In such situations, the prediction of nonlinear extrapolation is equivalent to that of linear extrapolation. Following this reasoning, a linear extrapolation conventionally considered as the basis or default (Mrotek and Soechting 2007a, b) in predicting target motion may instead be considered as the prediction in a special situation when a target moves at a constant velocity. This novel perspective may explain contradictions such as situational and individual differences in the prediction of target motion (Mrotek and Soechting 2007a, b).

On the basis of computational neuroscience studies, the existence of an internal model in the central nervous system has been established (Wolpert et al. 1998, 2011; Kawato 1999; Imamizu et al. 2000). In general, internal models are associated with predicting the motion of one's own body (e.g., arm) and tools, and can be used to maintain stability in the presence of feedback (or sensorimotor) delays when trying to make rapid movements under feedback control. On the other hand, some researchers have proposed that the notion of internal models can be extended to predicting the behavior of other persons (Wolpert et al. 2003), but there is little experimental evidence. Here, we have shown that neural network models were able to learn a flexible and accurate predictions that could be useful against unknown opponents, and these results suggest the feasibility of acquiring the “internal model of opponent motion,” especially in this type of interaction.

## Supplementary Information

Below is the link to the electronic supplementary material.

**Supplementary Fig. 1**. Unpredictability in target motion. Verification of the independence of the unpredictability (entropy) in target motion on the sampling frequency and bin width. We tested whether the unpredictability changes when the sampling frequency and the bin width are different, and confirmed that this　does not change qualitatively. (PDF 4576 kb)

**Supplementary Fig. 2**. Simple reaction task. **a** Illustration of the experimental task. Participants (n = 12) waited for the stimulus to be presented (foreperiod), and could tilt the joystick of a controller in any direction as soon as the stimulus (red disk) is presented (response time). The fore-period was randomly sampled from a Gaussian distribution with a mean of 3 and a variance of 1. The response was defined as the joystick being tilted by 80% or more of its maximum. Each participant made 50 trials. **b** Relative frequency distribution of response times. Response time was defined as the temporal differences between presentation of stimulus and response. Bin width was set to 50 ms. Data represent the mean ± s.e.m across participants. (PDF 4504 kb)

**Supplementary Fig. 3**. Relative frequency distribution of response times. Verification of the independence of the shape of the relative frequency distribution of the response times on the bin width. Response time was defined as the temporal difference between the target and pursuer in the zero-crossing of their horizontal velocities. We tested whether the relative frequency distribution of the response times changes when the bin width are different, and confirmed that this　does not change qualitatively. (PDF 4592 kb)

**Supplementary Fig. 4**. Prediction of target movement direction at the next time-step for phases with models. **a** Predicted relative frequency distribution of the difference in target moving direction between at time t and time *t* + 1 by models for each of the three experimental conditions. L, C, LN, NN, and RNN denote the linear, curvilinear, linear neural network, nonlinear neural network, and recurrent neural network models, respectively. Bin width was set to 5 degrees. Dashed lines denote the actual relative frequency distribution (same as mean across participants in Fig. 1e). **b** Angular error of the model prediction in each phase (straight (ST), gentle curve (GC), and sharp curve (SC)) for each experimental condition. The ST, GC, and SC were defined as the cases where differences in the target moving direction between at time *t*and time *t*+ 1 were less than 20, 20–60, and more than 60 degrees, respectively. Ring at the top of each panel denotes the mean proportions across participants among the phases. For all panels, quantitative data represent the mean ± s.e.m across participants. (PDF 4747 kb)

**Supplementary Fig. 5**. Prediction of target movement direction at the next time-step with models. **a** Illustration of neural network models. The models predicted the next state of attacker (target) using the current states of attacker and defender (pursuer). The long short-term memory (LSTM) model is a network that replaces the hidden layer of the RNN model with an LSTM layer (upper left panel). Deep nonlinear neural network (DNN), deep recurrent neural network (DRNN), and deep long short-term memory (DLSTM) models have two hidden layers each (upper right and lower panels). **b** Two-dimensional relative frequency distribution of predicted and actual directions of target movement for each experimental condition (pooled data for all participants). LSTM, DNN, DRNN, and DLSTM denote the long short-term memory, deep nonlinear neural network, deep recurrent neural network, and deep long short-term memory models, respectively. Bin width was set to 5 degrees. **c** Angular error of the model prediction for each of the experimental conditions. For all panels, quantitative data represent the mean ± s.e.m across participants. (PDF 5289 kb)

**Supplementary Fig. 6**. Prediction of target movement direction at the next time-step for phases with models. **a** Predicted relative frequency distribution of the difference in moving direction of the target between at time *t*and time *t* + 1 by models for each experimental condition. LSTM, DNN, DRNN, and DLSTM denote the long short-term memory, deep nonlinear neural network, deep recurrent neural network, and deep long short-term memory models, respectively. The width of each bin was set to 5 degrees. The dashed line denote the actual relative frequency distribution (same as mean across participants in Fig. 1d). **b** Angular error of the model prediction in each phase (straight, gentle curve, and sharp curve) for each of the experimental conditions. The straight (ST), gentle curve (GC), and sharp curve (SC) phases were defined as the case where differences in the moving direction of the target between time *t*and time *t* + 1 were less than 20, 20–60, and more than 60 degrees, respectively. Ring at the top of each panel denotes the mean proportions across participants among the phases. For all panels, quantitative data represent the mean ± s.e.m across participants. (PDF 4737 kb)

**Supplementary Fig. 7**. Sequential prediction of target motion with models. **a** Illustration of sequential prediction by neural network models. The predicted state of the attacker (target) was used as a part of model input at the next time-step, and the prediction was made sequentially up to 5 steps (250 ms) ahead. Assuming that the own state of defender (pursuer) could be accurately estimated by the internal model of own motion, we used the actual state of the defender as a part of model input for sequential prediction. The same procedure was used for all neural network models. **b** Examples of predicted and actual trajectories. LSTM, DNN, DRNN, and DLSTM denote the long short-term memory, deep nonlinear neural network, deep recurrent neural network, and deep long short-term memory models, respectively. Red and blue lines show the actual trajectories of the attacker and defender, respectively. The disks denote the end point of the predicted and actual trajectories. For ease of visibility, the trajectories of attacker and defender from the 10 time steps (500 ms) before, to the time of prediction start, are shown by the dashed red and blue lines. ST, GC, and SC denote, respectively, the straight, gentle curve, and sharp curve phases, representing the phase of each prediction at time-steps from *t* + 1 to *t* + 5. **c** Average displacement error of the sequential model prediction up to 5 time-steps (250 ms) ahead for each of the experimental conditions. **d** Final displacement error of the sequential model prediction up to 5 time-steps (250 ms) ahead. For all panels, quantitative data represent the mean ± s.e.m across participants. (PDF 4689 kb)

**Supplementary Fig. 8**. Frequency distribution of directional changes in interpersonal distance. Frequency distribution of time spent in each interpersonal distance (left), frequency distribution of directional changes in each interpersonal distance (middle), and frequency distribution per time of directional changes in each interpersonal distance (right), for each experimental condition. Bin width was set 1 mm. For all panels, quantitative data represent the mean ± s.e.m across participants. (PDF 4642 kb)

Supplementary file 9 (PDF 100 kb)

Supplementary file 10 (PDF 176 kb)

## Data Availability

The data supporting the findings of this study are available in figshare at https://doi.org/10.6084/m9.figshare.14405354.

## Code availability

The codes are available from the corresponding author upon reasonable request.
