# Supplementary figures and images for: Flexible prediction of opponent motion with internal representation in interception behavior
Source: Biol Cybern. 2021 Aug 11;115(5):473–85. doi: 10.1007/s00422-021-00891-9 (PMC8551111; doi:10.1007/s00422-021-00891-9)

20 Hz, bin width = 5 deg

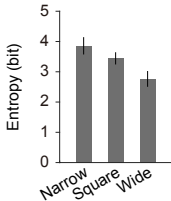

120 Hz, bin width = 5 deg

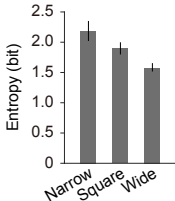

20 Hz, bin width = 1 deg

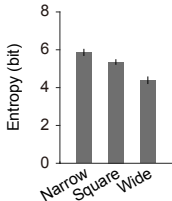

Supplement: Supplementary file 1 — Supplementary Fig. 1. Unpredictability in target motion. Verification of the independence of the unpredictability (entropy) in target motion on the sampling frequency and bin width. We tested whether the unpredictability changes when the sampling frequency and the bin width are different, and confirmed that this does not change qualitatively. (PDF 4576 kb) [file 422_2021_891_MOESM1_ESM.pdf]

**a**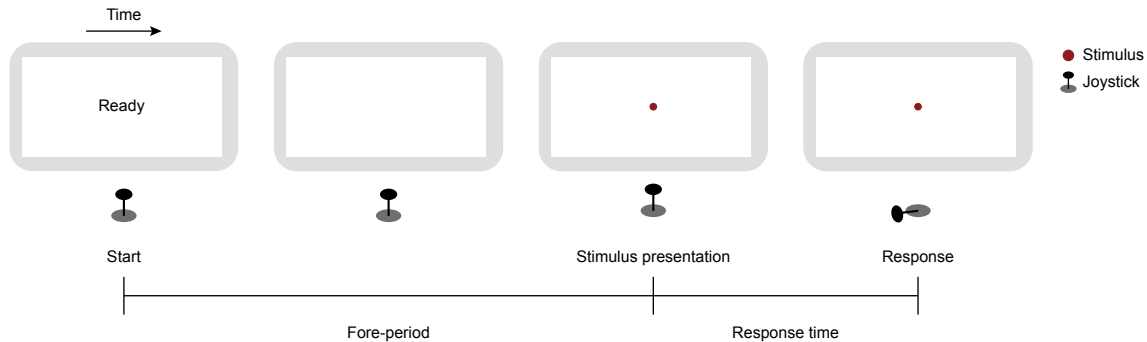**b**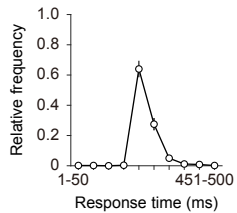

Supplement: Supplementary file 2 — Supplementary Fig. 2. Simple reaction task. a Illustration of the experimental task. Participants (n = 12) waited for the stimulus to be presented (foreperiod), and could tilt the joystick of a controller in any direction as soon as the stimulus (red disk) is presented (response time). The fore-period was randomly sampled from a Gaussian distribution with a mean of 3 and a variance of 1. The response was defined as the joystick being tilted by 80% or more of its maximum. Each participant made 50 trials. b Relative frequency distribution of response times. Response time was defined as the temporal differences between presentation of stimulus and response. Bin width was set to 50 ms. Data represent the mean ± s.e.m across participants. (PDF 4504 kb) [file 422_2021_891_MOESM2_ESM.pdf]

Bin width = 50 ms

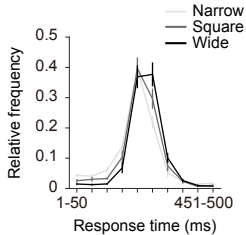

Bin width = 25 ms

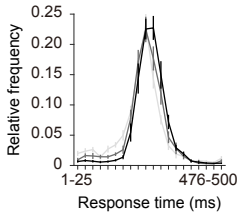

Bin width = 100 ms

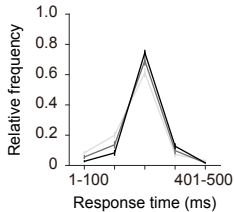

Supplement: Supplementary file 3 — Supplementary Fig. 3. Relative frequency distribution of response times. Verification of the independence of the shape of the relative frequency distribution of the response times on the bin width. Response time was defined as the temporal difference between the target and pursuer in the zero-crossing of their horizontal velocities. We tested whether the relative frequency distribution of the response times changes when the bin width are different, and confirmed that this does not change qualitatively. (PDF 4592 kb) [file 422_2021_891_MOESM3_ESM.pdf]

**a**

Narrow

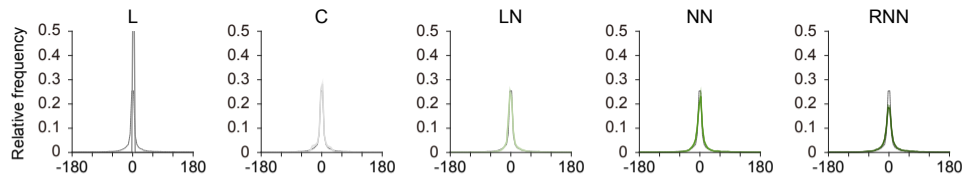

Square

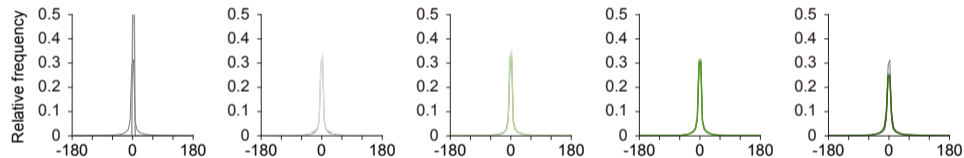

Wide

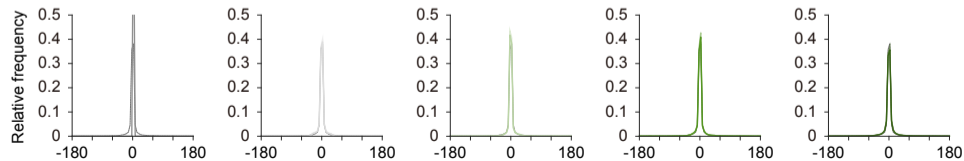

Difference from previous movement direction (deg)

**b**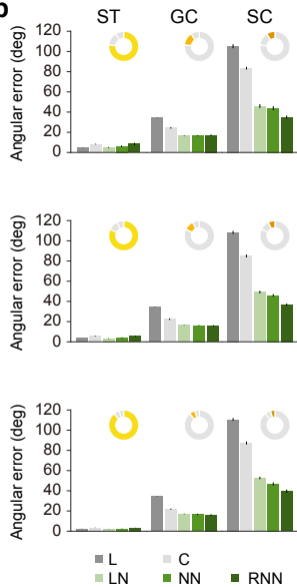

Supplement: Supplementary file 4 — Supplementary Fig. 4. Prediction of target movement direction at the next time-step for phases with models. a Predicted relative frequency distribution of the difference in target moving direction between at time t and time t + 1 by models for each of the three experimental conditions. L, C, LN, NN, and RNN denote the linear, curvilinear, linear neural network, nonlinear neural network, and recurrent neural network models, respectively. Bin width was set to 5 degrees. Dashed lines denote the actual relative frequency distribution (same as mean across participants in Fig. 1e). b Angular error of the model prediction in each phase (straight (ST), gentle curve (GC), and sharp curve (SC)) for each experimental condition. The ST, GC, and SC were defined as the cases where differences in the target moving direction between at time tand time t+ 1 were less than 20, 20–60, and more than 60 degrees, respectively. Ring at the top of each panel denotes the mean proportions across participants among the phases. For all panels, quantitative data represent the mean ± s.e.m across participants. (PDF 4747 kb) [file 422_2021_891_MOESM4_ESM.pdf]

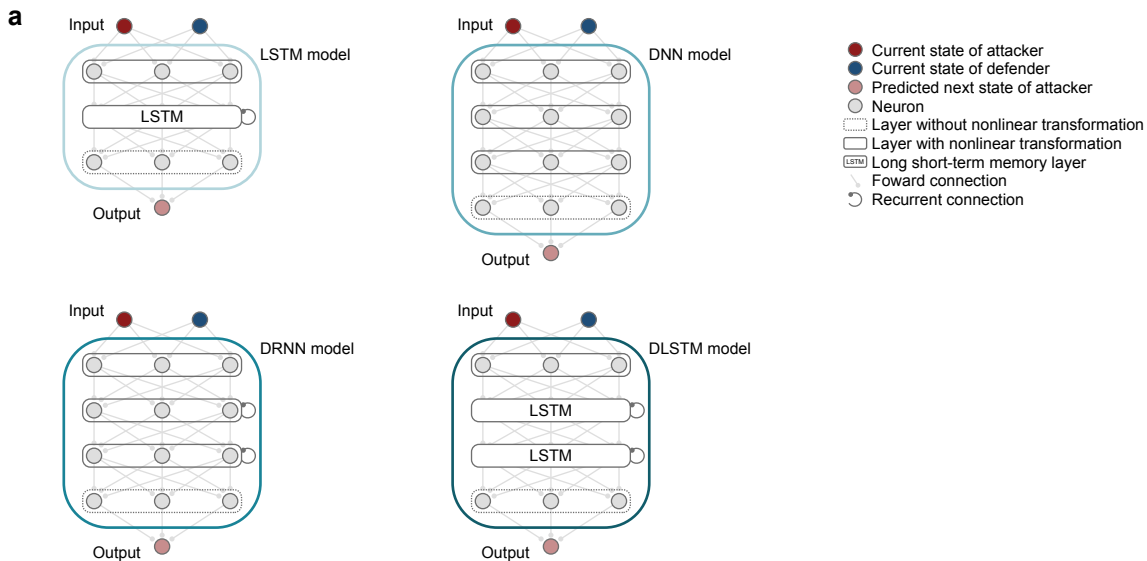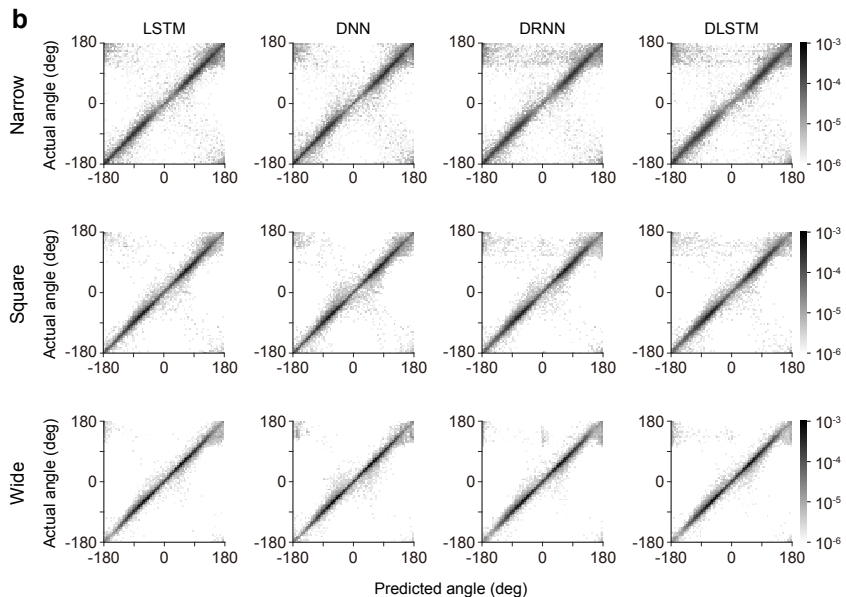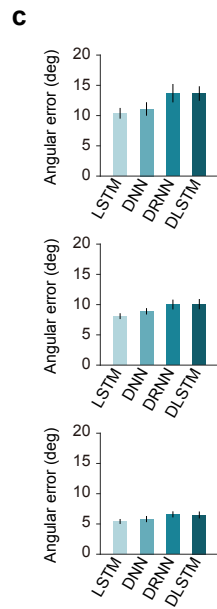

Supplement: Supplementary file 5 — Supplementary Fig. 5. Prediction of target movement direction at the next time-step with models. a Illustration of neural network models. The models predicted the next state of attacker (target) using the current states of attacker and defender (pursuer). The long short-term memory (LSTM) model is a network that replaces the hidden layer of the RNN model with an LSTM layer (upper left panel). Deep nonlinear neural network (DNN), deep recurrent neural network (DRNN), and deep long short-term memory (DLSTM) models have two hidden layers each (upper right and lower panels). b Two-dimensional relative frequency distribution of predicted and actual directions of target movement for each experimental condition (pooled data for all participants). LSTM, DNN, DRNN, and DLSTM denote the long short-term memory, deep nonlinear neural network, deep recurrent neural network, and deep long short-term memory models, respectively. Bin width was set to 5 degrees. c Angular error of the model prediction for each of the experimental conditions. For all panels, quantitative data represent the mean ± s.e.m across participants. (PDF 5289 kb) [file 422_2021_891_MOESM5_ESM.pdf]

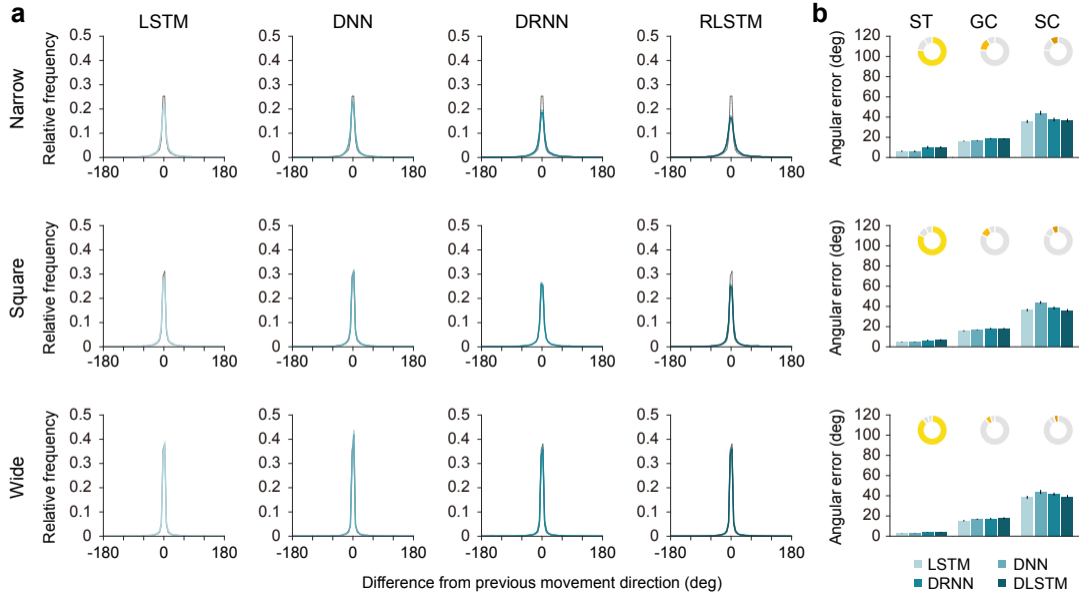

Supplement: Supplementary file 6 — Supplementary Fig. 6. Prediction of target movement direction at the next time-step for phases with models. a Predicted relative frequency distribution of the difference in moving direction of the target between at time tand time t + 1 by models for each experimental condition. LSTM, DNN, DRNN, and DLSTM denote the long short-term memory, deep nonlinear neural network, deep recurrent neural network, and deep long short-term memory models, respectively. The width of each bin was set to 5 degrees. The dashed line denote the actual relative frequency distribution (same as mean across participants in Fig. 1d). b Angular error of the model prediction in each phase (straight, gentle curve, and sharp curve) for each of the experimental conditions. The straight (ST), gentle curve (GC), and sharp curve (SC) phases were defined as the case where differences in the moving direction of the target between time tand time t + 1 were less than 20, 20–60, and more than 60 degrees, respectively. Ring at the top of each panel denotes the mean proportions across participants among the phases. For all panels, quantitative data represent the mean ± s.e.m across participants. (PDF 4737 kb) [file 422_2021_891_MOESM6_ESM.pdf]

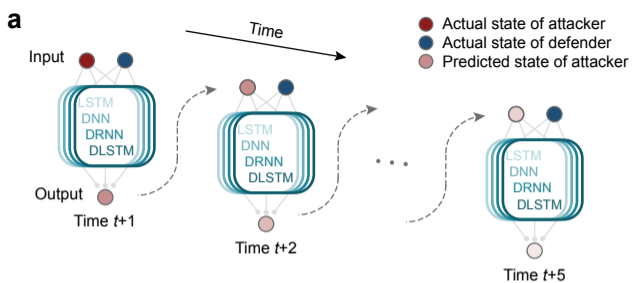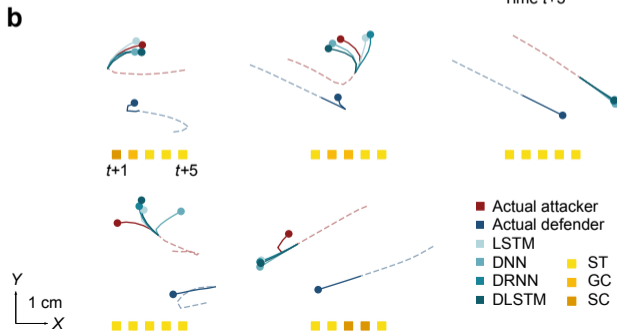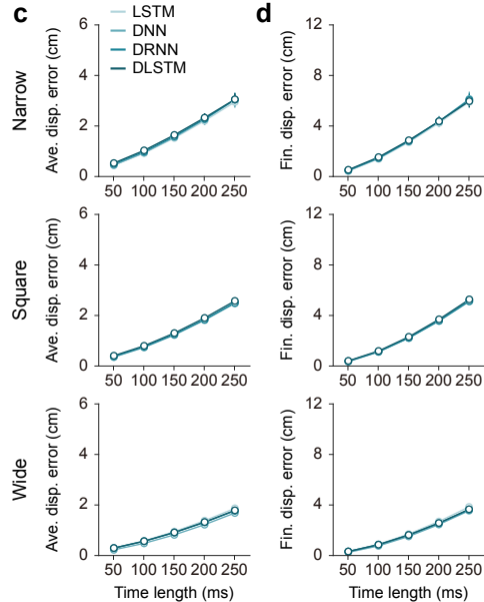

Supplement: Supplementary file 7 — Supplementary Fig. 7. Sequential prediction of target motion with models. a Illustration of sequential prediction by neural network models. The predicted state of the attacker (target) was used as a part of model input at the next time-step, and the prediction was made sequentially up to 5 steps (250 ms) ahead. Assuming that the own state of defender (pursuer) could be accurately estimated by the internal model of own motion, we used the actual state of the defender as a part of model input for sequential prediction. The same procedure was used for all neural network models. b Examples of predicted and actual trajectories. LSTM, DNN, DRNN, and DLSTM denote the long short-term memory, deep nonlinear neural network, deep recurrent neural network, and deep long short-term memory models, respectively. Red and blue lines show the actual trajectories of the attacker and defender, respectively. The disks denote the end point of the predicted and actual trajectories. For ease of visibility, the trajectories of attacker and defender from the 10 time steps (500 ms) before, to the time of prediction start, are shown by the dashed red and blue lines. ST, GC, and SC denote, respectively, the straight, gentle curve, and sharp curve phases, representing the phase of each prediction at time-steps from t + 1 to t + 5. c Average displacement error of the sequential model prediction up to 5 time-steps (250 ms) ahead for each of the experimental conditions. d Final displacement error of the sequential model prediction up to 5 time-steps (250 ms) ahead. For all panels, quantitative data represent the mean ± s.e.m across participants. (PDF 4689 kb) [file 422_2021_891_MOESM7_ESM.pdf]

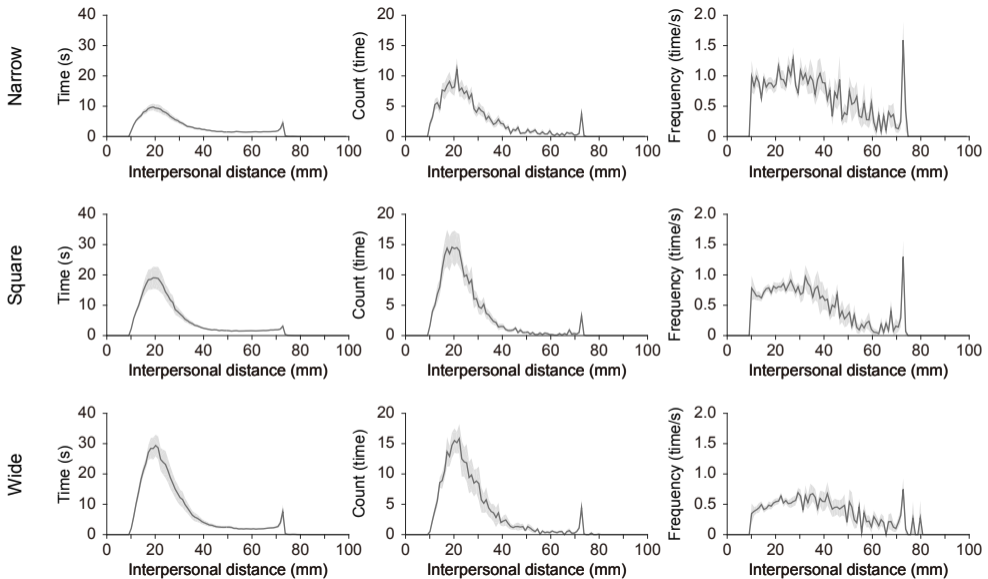

Supplement: Supplementary file 8 — Supplementary Fig. 8. Frequency distribution of directional changes in interpersonal distance. Frequency distribution of time spent in each interpersonal distance (left), frequency distribution of directional changes in each interpersonal distance (middle), and frequency distribution per time of directional changes in each interpersonal distance (right), for each experimental condition. Bin width was set 1 mm. For all panels, quantitative data represent the mean ± s.e.m across participants. (PDF 4642 kb) [file 422_2021_891_MOESM8_ESM.pdf]
