# Supplementary material for: Flexible prediction of opponent motion with internal representation in interception behavior
Source: Biol Cybern. 2021 Aug 11;115(5):473–85. doi: 10.1007/s00422-021-00891-9 (PMC8551111; doi:10.1007/s00422-021-00891-9)
Supplement: Supplementary file 9 — (PDF 100 kb) [file 422_2021_891_MOESM9_ESM.pdf]

Supplementary Table 1

Possible and used values of hyperparameters for neural network models.

Description	Possible values	Used value
Learning rate	{0.3, 0.03, 0.003, 0.0003, 0.00003}	0.0003
Batch size	{16, 32, 64, 128}	16
