# Supplementary material for: Flexible prediction of opponent motion with internal representation in interception behavior
Source: Biol Cybern. 2021 Aug 11;115(5):473–85. doi: 10.1007/s00422-021-00891-9 (PMC8551111; doi:10.1007/s00422-021-00891-9)
Supplement: Supplementary file 10 — (PDF 176 kb) [file 422_2021_891_MOESM10_ESM.pdf]

Supplementary Table 2

Comprehensive statistical information of post-hoc comparisons.

Figure	Test	Comparison	t-value	P-value
Fig. 2b	Holm-Bonferroni method for condition	narrow vs. square	5.8347	0.0001
		narrow vs. wide	11.5332	0.0000
		square vs. wide	8.7454	0.0000
Fig. 3c	Holm-Bonferroni method for condition	narrow vs. square	3.0119	0.0236
		narrow vs. wide	5.6854	0.0004
		square vs. wide	3.0135	0.0236
Fig. 3e	Holm-Bonferroni method for horizontal position at narrow	-	-	-
	Holm-Bonferroni method for horizontal position at square	-	-	-
	Holm-Bonferroni method for horizontal position at wide	column 1 vs. 2	2.0950	1.0000
		column 1 vs. 3	0.6881	1.0000
		column 1 vs. 4	1.3826	1.0000
		column 1 vs. 5	0.2779	1.0000
		column 1 vs. 6	0.0699	1.0000
		column 1 vs. 7	3.5683	0.1146
		column 1 vs. 8	0.0083	1.0000
		column 2 vs. 3	0.8823	1.0000
		column 2 vs. 4	0.1599	1.0000
		column 2 vs. 5	1.6805	1.0000
		column 2 vs. 6	2.1603	1.0000
		column 2 vs. 7	1.9384	1.0000
		column 2 vs. 8	2.2792	1.0000
		column 3 vs. 4	1.2511	1.0000
		column 3 vs. 5	0.4788	1.0000
		column 3 vs. 6	0.7261	1.0000

		column 3 vs. 7	2.6100	0.5822
		column 3 vs. 8	0.4469	1.0000
		column 4 vs. 5	1.3781	1.0000
		column 4 vs. 6	1.5312	1.0000
		column 4 vs. 7	1.6962	1.0000
		column 4 vs. 8	1.3138	1.0000
		column 5 vs. 6	0.4698	1.0000
		column 5 vs. 7	3.7484	0.0901
		column 5 vs. 8	0.1862	1.0000
		column 6 vs. 7	3.5936	0.1139
		column 6 vs. 8	0.0834	1.0000
		column 7 vs. 8	3.2180	0.2047
Fig. 3g	Holm-Bonferroni method for condition	narrow vs. square	4.1239	0.0017
		narrow vs. wide	9.9657	0.0000
		square vs. wide	8.5445	0.0000
Fig. 4c	Holm-Bonferroni method for condition	narrow vs. square	5.2813	0.0003
		narrow vs. wide	9.2231	0.0000
		square vs. wide	10.9090	0.0000
	Holm-Bonferroni method for model	L vs. C	4.3006	0.0050
		L vs. LN	21.7650	0.0000
		L vs. NN	15.2058	0.0000
		L vs. RNN	10.7151	0.0000
		C vs. LN	17.4482	0.0000
		C vs. NN	11.7516	0.0000
		C vs. RNN	9.2195	0.0000
		LN vs. NN	2.0924	0.0604
		LN vs. RNN	3.6971	0.0106
		NN vs. RNN	3.2511	0.0154
	Holm-Bonferroni method for condition at L ($F_{2, 22} = 60.4908$, $P < 0.0001$)	L: narrow vs. square	4.6082	0.0000
		L: narrow vs. wide	8.9156	0.0000

	L: square vs. wide	8.8074	0.0008
Holm-Bonferroni method for condition at C ($F_{2, 22}$ = 82.3925, P < 0.0001)	C: narrow vs. square	5.7816	0.0001
	C: narrow vs. wide	11.0136	0.0000
	C: square vs. wide	9.0884	0.0000
Holm-Bonferroni method for condition at LN ($F_{1.22, 13.38}$ = 45.8426, P < 0.0001)	LN: narrow vs. square	4.6302	0.0007
	LN: narrow vs. wide	7.3127	0.0000
	LN: square vs. wide	8.8312	0.0000
Holm-Bonferroni method for condition at NN ($F_{1.26, 13.83}$ = 39.6637, P < 0.0001)	NN: narrow vs. square	4.1546	0.0016
	NN: narrow vs. wide	7.1126	0.0000
	NN: square vs. wide	8.5533	0.0000
Holm-Bonferroni method for condition at RNN ($F_{1.09, 12.03}$ = 36.8165, P < 0.0001)	RNN: narrow vs. square	4.6569	0.0007
	RNN: narrow vs. wide	6.3317	0.0001
	RNN: square vs. wide	8.0298	0.0000
Holm-Bonferroni method for model at narrow ($F_{1.41, 15.53}$ = 66.4812, P < 0.0001)	narrow: L vs. C	1.4241	0.1822
	narrow: L vs. LN	14.2016	0.0000
	narrow: L vs. NN	5.2058	0.0000
	narrow: L vs. RNN	5.9387	0.0006
	narrow: C vs. LN	14.1349	0.0000

		narrow: C vs. NN	9.4688	0.0000
		narrow: C vs. RNN	5.8484	0.0006
		narrow: LN vs. NN	2.7025	0.0617
		narrow: LN vs. RNN	2.9820	0.0499
		narrow: NN vs. RNN	2.2152	0.0976
	Holm-Bonferroni method for model at square ($F_{1.68, 18.47}$ = 117.7832, P < 0.0001)	square: L vs. C	4.1662	0.0063
		square: L vs. LN	17.2371	0.0000
		square: L vs. NN	13.8059	0.0000
		square: L vs. RNN	11.1687	0.0000
		square: C vs. LN	11.1555	0.0000
		square: C vs. NN	9.3351	0.0000
		square: C vs. RNN	8.5302	0.0000
		square: LN vs. NN	1.4410	0.1774
		square: LN vs. RNN	3.7460	0.0097
		square: NN vs. RNN	2.8725	0.0303
	Holm-Bonferroni method for model at wide ($F_{1.91, 21.06}$ = 158.0900, P < 0.0001)	wide: L vs. C	5.6912	0.0006
		wide: L vs. LN	16.0002	0.0000
		wide: L vs. NN	16.3802	0.0000
		wide: L vs. RNN	11.5406	0.0000
		wide: C vs. LN	18.3121	0.0000
		wide: C vs. NN	12.3197	0.0000
		wide: C vs. RNN	11.4491	0.0000
		wide: LN vs. NN	0.8759	0.3998
		wide: LN vs. RNN	4.1872	0.0046
		wide: NN vs. RNN	1.8510	0.1824
Fig. 5c	Holm-Bonferroni method for phase	narrow vs. square	2.2513	0.0458
		narrow vs. wide	5.1279	0.0007
		square vs. wide	11.0171	0.0000
	Holm-Bonferroni method for time length	50 vs. 100	20.0908	0.0000
		50 vs. 150	20.5218	0.0000
		50 vs. 200	21.0972	0.0000
		50 vs. 250	21.7350	0.0000

	100 vs. 150	20.8639	0.0000
	100 vs. 200	21.4727	0.0000
	100 vs. 250	22.1266	0.0000
	150 vs. 200	22.0070	0.0000
	150 vs. 250	22.6601	0.0000
	200 vs. 250	23.2596	0.0000
Holm-Bonferroni method for model	L vs. C	20.2288	0.0000
	L vs. LN	7.3585	0.0001
	L vs. NN	12.8209	0.0000
	L vs. RNN	10.2927	0.0000
	C vs. LN	5.1320	0.0007
	C vs. NN	10.2812	0.0000
	C vs. RNN	7.8981	0.0000
	LN vs. NN	5.9915	0.0004
	LN vs. RNN	4.2101	0.0015
	NN vs. RNN	5.4731	0.0006
Holm-Bonferroni method for condition at 50	50: narrow vs. square	2.5090	0.0290
($F_{1.11, 12.21} = 21.1788$, $P < 0.001$)	50: narrow vs. wide	5.2306	0.0006
	50: square vs. wide	10.7129	0.0000
Holm-Bonferroni method for condition at 100	100: narrow vs. square	2.4494	0.0323
($F_{1.11, 12.24} = 21.3539$, $P < 0.001$)	100: narrow vs. wide	5.2189	0.0006
	100: square vs. wide	10.7857	0.0000
Holm-Bonferroni method for condition at 150	150: narrow vs. square	2.3408	0.0391
($F_{1.11, 12.26} = 21.1990$, $P < 0.001$)	150: narrow vs. wide	5.1721	0.0006

	150: square vs. wide	10.8986	0.0000
Holm-Bonferroni method for condition at 200 ($F_{1.12, 12.28}$ = 20.9690, P < 0.001)	200: narrow vs. square	2.2203	0.0483
	200: narrow vs. wide	5.1128	0.0007
	200: square vs. wide	10.9816	0.0000
Holm-Bonferroni method for condition at 250 ($F_{1.12, 12.28}$ = 20.6956, P < 0.001)	250: narrow vs. square	2.0850	0.0612
	250: narrow vs. wide	5.0467	0.0007
	250: square vs. wide	11.1609	0.0000
Holm-Bonferroni method for time length at narrow ($F_{1.00, 11.02}$ = 131.1018, P < 0.001)	narrow: 50 vs. 100	10.8487	0.0000
	narrow: 50 vs. 150	11.0235	0.0000
	narrow: 50 vs. 200	11.2417	0.0000
	narrow: 50 vs. 250	11.4568	0.0000
	narrow: 100 vs. 150	11.1641	0.0000
	narrow: 100 vs. 200	11.3916	0.0000
	narrow: 100 vs. 250	11.6052	0.0000
	narrow: 150 vs. 200	11.5932	0.0000
	narrow: 150 vs. 250	11.7940	0.0000
	narrow: 200 vs. 250	11.9802	0.0000
Holm-Bonferroni method for time length at square ($F_{1.01, 11.06}$ = 665.3718, P < 0.001)	square : 50 vs. 100	23.8545	0.0000
	square : 50 vs. 150	24.3558	0.0000
	square : 50 vs. 200	25.0561	0.0000
	square : 50 vs. 250	25.8519	0.0000
	square : 100 vs. 150	24.7411	0.0000
	square : 100 vs. 200	25.4825	0.0000
	square : 100 vs. 250	26.2919	0.0000
	square : 150 vs. 200	26.1076	0.0000

	square : 150 vs. 250	26.9068	0.0000
	square : 200 vs. 250	27.5855	0.0000
Holm-Bonferroni method for time length at wide ($F_{1.00, 11.05}$ = 296.8878, P < 0.001)	wide: 50 vs. 100	15.8689	0.0000
	wide: 50 vs. 150	16.2837	0.0000
	wide: 50 vs. 200	16.6919	0.0000
	wide: 50 vs. 250	17.2669	0.0000
	wide: 100 vs. 150	16.6123	0.0000
	wide: 100 vs. 200	16.9851	0.0000
	wide: 100 vs. 250	17.5787	0.0000
	wide: 150 vs. 200	17.2865	0.0000
	wide: 150 vs. 250	17.9566	0.0000
	wide: 200 vs. 250	18.5669	0.0000
Holm-Bonferroni method for condition at L ($F_{1.15, 12.61}$ = 15.9555, P = 0.001)	L: narrow vs. square	1.8832	0.0864
	L: narrow vs. wide	4.4741	0.0019
	L: square vs. wide	8.8631	0.0000
Holm-Bonferroni method for condition at C ($F_{1.14, 12.50}$ = 15.6676, P = 0.001)	C: narrow vs. square	2.0691	0.0629
	C: narrow vs. wide	4.3947	0.0021
	C: square vs. wide	8.3074	0.0000
Holm-Bonferroni method for condition at LN ($F_{1.30, 14.34}$ = 102.1775, P < 0.001)	LN: narrow vs. square	4.8760	0.0005
	LN: narrow vs. wide	11.5034	0.0000
	LN: square vs. wide	16.3001	0.0000
Holm-Bonferroni method for condition at NN	NN: narrow vs. square	2.1390	0.0557

($F_{1.06, 11.67}$ = 16.8278, P = 0.001)	NN: narrow vs. wide	4.6266	0.0015
	NN: square vs. wide	12.7631	0.0000
Holm-Bonferroni method for condition at RNN	RNN: narrow vs. square	1.8912	0.0852
($F_{1.05, 11.53}$ = 15.4477, P = 0.002)	RNN: narrow vs. wide	4.4262	0.0020
	RNN: square vs. wide	14.4759	0.0000
Holm-Bonferroni method	narrow: L vs. C	10.2181	0.0000
for model at narrow	narrow: L vs. LN	3.7884	0.0134
($F_{1.10, 12.10}$ = 27.1546,	narrow: L vs. NN	7.3648	0.0001
P < 0.001)	narrow: L vs. RNN	6.6515	0.0003
	narrow: C vs. LN	2.8961	0.0291
	narrow: C vs. NN	6.6365	0.0003
	narrow: C vs. RNN	5.8118	0.0007
	narrow: LN vs. NN	3.4016	0.0177
	narrow: LN vs. RNN	3.8535	0.0134
	narrow: NN vs. RNN	0.7875	0.4477
Holm-Bonferroni method	square: L vs. C	11.0824	0.0000
for model at square	square: L vs. LN	8.3974	0.0000
($F_{1.19, 13.14}$ = 79.3749,	square: L vs. NN	11.4873	0.0000
P < 0.001)	square: L vs. RNN	9.5287	0.0000
	square: C vs. LN	5.6817	0.0003
	square: C vs. NN	9.4084	0.0000
	square: C vs. RNN	7.4582	0.0001
	square: LN vs. NN	9.3872	0.0000
	square: LN vs. RNN	6.8261	0.0001
	square: NN vs. RNN	5.6657	0.0003
Holm-Bonferroni method	wide: L vs. C	9.2063	0.0000
for model at wide	wide: L vs. LN	8.3802	0.0000

($F_{1.25, 13.70}$ = 65.8659,	wide: L vs. NN	11.7481	0.0000
P < 0.001)	wide: L vs. RNN	6.7346	0.0002
	wide: C vs. LN	6.7084	0.0002
	wide: C vs. NN	10.4357	0.0000
	wide: C vs. RNN	4.9884	0.0012
	wide: LN vs. NN	3.8197	0.0028
	wide: LN vs. RNN	4.5029	0.0018
	wide: NN vs. RNN	11.5556	0.0000
Holm-Bonferroni method	L: 50 vs. 100	17.2526	0.0000
for time length at L	L: 50 vs. 150	17.4156	0.0000
($F_{1.00, 11.03}$ = 331.3701,	L: 50 vs. 200	17.8162	0.0000
P < 0.001)	L: 50 vs. 250	18.2363	0.0000
	L: 100 vs. 150	17.5354	0.0000
	L: 100 vs. 200	18.0341	0.0000
	L: 100 vs. 250	18.4829	0.0000
	L: 150 vs. 200	18.4889	0.0000
	L: 150 vs. 250	18.8964	0.0000
	L: 200 vs. 250	19.2644	0.0000
Holm-Bonferroni method	C: 50 vs. 100	15.4413	0.0000
for time length at C	C: 50 vs. 150	15.9191	0.0000
($F_{1.00, 11.03}$ = 288.0098,	C: 50 vs. 200	16.4392	0.0000
P < 0.001)	C: 50 vs. 250	16.9780	0.0000
	C: 100 vs. 150	16.3254	0.0000
	C: 100 vs. 200	16.8491	0.0000
	C: 100 vs. 250	17.3851	0.0000
	C: 150 vs. 200	17.3234	0.0000
	C: 150 vs. 250	17.8471	0.0000
	C: 200 vs. 250	18.3297	0.0000
Holm-Bonferroni method	LN: 50 vs. 100	37.2000	0.0000
for time length at LN	LN: 50 vs. 150	38.9802	0.0000
($F_{1.03, 11.3}$ = 1844.2486,	LN: 50 vs. 200	40.8283	0.0000
P < 0.001)	LN: 50 vs. 250	43.4381	0.0000

	LN: 100 vs. 150	40.2856	0.0000
	LN: 100 vs. 200	41.8700	0.0000
	LN: 100 vs. 250	44.4992	0.0000
	LN: 150 vs. 200	42.6543	0.0000
	LN: 150 vs. 250	45.6432	0.0000
	LN: 200 vs. 250	48.2883	0.0000
Holm-Bonferroni method for time length at NN ($F_{1.00, 11.05}$ = 374.8485, $P < 0.001$)	NN: 50 vs. 100	17.7578	0.0000
	NN: 50 vs. 150	18.1307	0.0000
	NN: 50 vs. 200	18.7964	0.0000
	NN: 50 vs. 250	19.3762	0.0000
	NN: 100 vs. 150	18.4187	0.0000
	NN: 100 vs. 200	19.1744	0.0000
	NN: 100 vs. 250	19.7406	0.0000
	NN: 150 vs. 200	19.8137	0.0000
	NN: 150 vs. 250	20.2581	0.0000
	NN: 200 vs. 250	20.6166	0.0000
Holm-Bonferroni method for time length at RNN ($F_{1.01, 11.12}$ = 469.8112, $P < 0.001$)	RNN: 50 vs. 100	21.0221	0.0000
	RNN: 50 vs. 150	21.0681	0.0000
	RNN: 50 vs. 200	21.3823	0.0000
	RNN: 50 vs. 250	21.8002	0.0000
	RNN: 100 vs. 150	21.0185	0.0000
	RNN: 100 vs. 200	21.3870	0.0000
	RNN: 100 vs. 250	21.8296	0.0000
	RNN: 150 vs. 200	21.5925	0.0000
	RNN: 150 vs. 250	22.0365	0.0000
	RNN: 200 vs. 250	22.3831	0.0000
Holm-Bonferroni method for model at 50 ($F_{1.07, 11.78}$ = 107.0474, $P < 0.001$)	50: L vs. C	24.7531	0.0000
	50: L vs. LN	10.0645	0.0000
	50: L vs. NN	13.7400	0.0000
	50: L vs. RNN	10.8060	0.0000
	50: C vs. LN	7.9149	0.0000
	50: C vs. NN	11.4003	0.0000

	50: C vs. RNN	8.6190	0.0000
	50: LN vs. NN	5.2356	0.0006
	50: LN vs. RNN	0.5642	0.5840
	50: NN vs. RNN	8.2598	0.0000
Holm-Bonferroni method for model at 100 ($F_{1.12, 12.34}$ = 91.2803, $P < 0.001$)	100: L vs. C	22.0075	0.0000
	100: L vs. LN	8.8093	0.0000
	100: L vs. NN	13.4164	0.0000
	100: L vs. RNN	10.5351	0.0000
	100: C vs. LN	6.4435	0.0002
	100: C vs. NN	10.7725	0.0000
	100: C vs. RNN	8.1066	0.0000
	100: LN vs. NN	6.0926	0.0002
	100: LN vs. RNN	3.2742	0.0074
	100: NN vs. RNN	6.2626	0.0002
Holm-Bonferroni method for model at 150 ($F_{1.17, 12.87}$ = 78.1474, $P < 0.001$)	150: L vs. C	21.1979	0.0000
	150: L vs. LN	7.6523	0.0001
	150: L vs. NN	12.8352	0.0000
	150: L vs. RNN	10.2082	0.0000
	150: C vs. LN	5.3298	0.0006
	150: C vs. NN	10.2393	0.0000
	150: C vs. RNN	7.7734	0.0001
	150: LN vs. NN	6.1684	0.0003
	150: LN vs. RNN	4.2150	0.0014
	150: NN vs. RNN	5.4460	0.0006
Holm-Bonferroni method for model at 200 ($F_{1.21, 13.31}$ = 69.5644, $P < 0.001$)	200: L vs. C	19.4186	0.0000
	200: L vs. LN	6.8026	0.0001
	200: L vs. NN	12.4223	0.0000
	200: L vs. RNN	10.0697	0.0000
	200: C vs. LN	4.5770	0.0016
	200: C vs. NN	9.8948	0.0000
	200: C vs. RNN	7.6607	0.0001
	200: LN vs. NN	6.1314	0.0003

	200: LN vs. RNN	4.5768	0.0016
	200: NN vs. RNN	5.0350	0.0011
Holm-Bonferroni method for model at 250 ($F_{1.24, 13.61}$ = 63.0106, P < 0.001)	250: L vs. C	17.5985	0.0000
	250: L vs. LN	6.1162	0.0004
	250: L vs. NN	12.1738	0.0000
	250: L vs. RNN	10.0001	0.0000
	250: C vs. LN	4.0214	0.0021
	250: C vs. NN	9.7500	0.0000
	250: C vs. RNN	7.6724	0.0001
	250: LN vs. NN	5.8215	0.0005
	250: LN vs. RNN	4.6547	0.0021
	250: NN vs. RNN	4.5749	0.0021
Holm-Bonferroni method for model at narrow and 50 ($F_{1.04, 11.46}$ = 36.6509, P < 0.001)	narrow, 50: L vs. C	11.1229	0.0000
	narrow, 50: L vs. LN	5.2279	0.0014
	narrow, 50: L vs. NN	7.6252	0.0001
	narrow, 50: L vs. RNN	6.6836	0.0002
	narrow, 50: C vs. LN	4.4752	0.0038
	narrow, 50: C vs. NN	6.9892	0.0002
	narrow, 50: C vs. RNN	5.9768	0.0006
	narrow, 50: LN vs. NN	3.4846	0.0153
	narrow, 50: LN vs. RNN	2.9809	0.0250
	narrow, 50: NN vs. RNN	2.4109	0.0346
Holm-Bonferroni method for model at narrow and 100 ($F_{1.07, 11.81}$ = 32.6881, P < 0.001)	narrow, 100: L vs. C	10.5590	0.0000
	narrow, 100: L vs. LN	4.5405	0.0042
	narrow, 100: L vs. NN	7.5840	0.0001
	narrow, 100: L vs. RNN	6.7304	0.0002
	narrow, 100: C vs. LN	3.6760	0.0100
	narrow, 100: C vs. NN	6.8963	0.0002
	narrow, 100: C vs. RNN	5.9405	0.0006
	narrow, 100: LN vs. NN	3.7616	0.0100
	narrow, 100: LN vs. RNN	3.8920	0.0100
	narrow, 100: NN vs. RNN	1.5462	0.1503

Holm-Bonferroni method for model at narrow and 150 ($F_{1.10, 12.14} = 28.3713$, $P < 0.001$)	narrow, 150: L vs. C	10.2464	0.0000
	narrow, 150: L vs. LN	3.9412	0.0101
	narrow, 150: L vs. NN	7.3717	0.0001
	narrow, 150: L vs. RNN	6.6482	0.0003
	narrow, 150: C vs. LN	3.0229	0.0232
	narrow, 150: C vs. NN	6.6540	0.0003
	narrow, 150: C vs. RNN	5.8110	0.0007
	narrow, 150: LN vs. NN	3.6424	0.0116
	narrow, 150: LN vs. RNN	4.0197	0.0101
	narrow, 150: NN vs. RNN	1.0214	0.3290
Holm-Bonferroni method for model at narrow and 200 ($F_{1.12, 12.32} = 25.0914$, $P < 0.001$)	narrow, 200: L vs. C	10.2206	0.0000
	narrow, 200: L vs. LN	3.4999	0.0199
	narrow, 200: L vs. NN	7.2232	0.0002
	narrow, 200: L vs. RNN	6.6026	0.0003
	narrow, 200: C vs. LN	2.5814	0.0511
	narrow, 200: C vs. NN	6.4544	0.0003
	narrow, 200: C vs. RNN	5.7218	0.0008
	narrow, 200: LN vs. NN	3.4044	0.0199
	narrow, 200: LN vs. RNN	3.9395	0.0116
	narrow, 200: NN vs. RNN	0.5440	0.5973
Holm-Bonferroni method for model at narrow and 250 ($F_{1.13, 12.41} = 22.4931$, $P < 0.001$)	narrow, 250: L vs. C	9.5456	0.0000
	narrow, 250: L vs. LN	3.1634	0.0361
	narrow, 250: L vs. NN	7.1229	0.0002
	narrow, 250: L vs. RNN	6.5470	0.0003
	narrow, 250: C vs. LN	2.2640	0.0896
	narrow, 250: C vs. NN	6.3470	0.0004
	narrow, 250: C vs. RNN	5.6547	0.0009
	narrow, 250: LN vs. NN	3.1408	0.0361
	narrow, 250: LN vs. RNN	3.7735	0.0154
	narrow, 250: NN vs. RNN	0.1424	0.8893
Holm-Bonferroni method for model at square and 50	square, 50: L vs. C	13.3844	0.0000
	square, 50: L vs. LN	11.1825	0.0000

($F_{1.10, 12.11}$ = 115.5001,	square, 50: L vs. NN	13.4958	0.0000
$P < 0.001$)	square, 50: L vs. RNN	10.9561	0.0000
	square, 50: C vs. LN	8.5428	0.0000
	square, 50: C vs. NN	11.1463	0.0000
	square, 50: C vs. RNN	8.5898	0.0000
	square, 50: LN vs. NN	7.3405	0.0000
	square, 50: LN vs. RNN	2.2000	0.0501
	square, 50: NN vs. RNN	9.3200	0.0000
Holm-Bonferroni method	square, 100: L vs. C	11.9442	0.0000
for model at square and	square, 100: L vs. LN	9.7474	0.0000
100	square, 100: L vs. NN	12.4398	0.0000
($F_{1.14, 12.57}$ = 93.2465,	square, 100: L vs. RNN	10.0873	0.0000
$P < 0.001$)	square, 100: C vs. LN	6.9117	0.0001
	square, 100: C vs. NN	10.0441	0.0000
	square, 100: C vs. RNN	7.7407	0.0000
	square, 100: LN vs. NN	8.8901	0.0000
	square, 100: LN vs. RNN	5.5415	0.0002
	square, 100: NN vs. RNN	6.7765	0.0001
Holm-Bonferroni method	square, 150: L vs. C	11.3869	0.0000
for model at square and	square, 150: L vs. LN	8.5636	0.0000
150	square, 150: L vs. NN	11.5935	0.0000
($F_{1.18, 12.98}$ = 79.6204,	square, 150: L vs. RNN	9.4909	0.0000
$P < 0.001$)	square, 150: C vs. LN	5.7664	0.0003
	square, 150: C vs. NN	9.3578	0.0000
	square, 150: C vs. RNN	7.2897	0.0001
	square, 150: LN vs. NN	9.2782	0.0000
	square, 150: LN vs. RNN	6.8113	0.0001
	square, 150: NN vs. RNN	5.7058	0.0003
Holm-Bonferroni method	square, 200: L vs. C	10.6850	0.0000
for model at square and	square, 200: L vs. LN	7.7849	0.0000
200	square, 200: L vs. NN	10.9330	0.0000
($F_{1.21, 13.36}$ = 72.0122,	square, 200: L vs. RNN	9.1191	0.0000

P < 0.001)	square, 200: C vs. LN	5.0883	0.0006
	square, 200: C vs. NN	8.9507	0.0000
	square, 200: C vs. RNN	7.1442	0.0001
	square, 200: LN vs. NN	9.6500	0.0000
	square, 200: LN vs. RNN	7.2388	0.0001
	square, 200: NN vs. RNN	5.1389	0.0006
Holm-Bonferroni method for model at square and 250 ($F_{1.26, 13.85}$ = 67.3528, P < 0.001)	square, 250: L vs. C	9.9700	0.0000
	square, 250: L vs. LN	7.1945	0.0001
	square, 250: L vs. NN	10.5553	0.0000
	square, 250: L vs. RNN	8.9118	0.0000
	square, 250: C vs. LN	4.5946	0.0015
	square, 250: C vs. NN	8.7889	0.0000
	square, 250: C vs. RNN	7.1468	0.0001
	square, 250: LN vs. NN	9.5101	0.0000
	square, 250: LN vs. RNN	7.2028	0.0001
	square, 250: NN vs. RNN	4.4993	0.0015
Holm-Bonferroni method for model at wide and 50 ($F_{1.09, 11.98}$ = 86.2249, P < 0.001)	wide, 50: L vs. C	9.7044	0.0000
	wide, 50: L vs. LN	9.7098	0.0000
	wide, 50: L vs. NN	11.9279	0.0000
	wide, 50: L vs. RNN	7.5432	0.0000
	wide, 50: C vs. LN	8.6872	0.0000
	wide, 50: C vs. NN	11.0902	0.0000
	wide, 50: C vs. RNN	6.1404	0.0001
	wide, 50: LN vs. NN	1.8254	0.0952
	wide, 50: LN vs. RNN	12.4277	0.0000
	wide, 50: NN vs. RNN	17.9134	0.0000
Holm-Bonferroni method for model at wide and 100 ($F_{1.14, 12.54}$ = 76.4186, P < 0.001)	wide, 100: L vs. C	9.9250	0.0000
	wide, 100: L vs. LN	9.1017	0.0000
	wide, 100: L vs. NN	11.9663	0.0000
	wide, 100: L vs. RNN	7.1918	0.0001
	wide, 100: C vs. LN	7.6861	0.0000
	wide, 100: C vs. NN	10.8166	0.0000

	wide, 100: C vs. RNN	5.5255	0.0004
	wide, 100: LN vs. NN	3.1845	0.0087
	wide, 100: LN vs. RNN	7.5977	0.0000
	wide, 100: NN vs. RNN	13.9133	0.0000
Holm-Bonferroni method for model at wide and 150 ($F_{1.21, 13.34} = 67.6345$, $P < 0.001$)	wide, 150: L vs. C	9.5282	0.0000
	wide, 150: L vs. LN	8.5429	0.0000
	wide, 150: L vs. NN	11.8345	0.0000
	wide, 150: L vs. RNN	6.8136	0.0001
	wide, 150: C vs. LN	6.8551	0.0001
	wide, 150: C vs. NN	10.4539	0.0000
	wide, 150: C vs. RNN	5.0262	0.0012
	wide, 150: LN vs. NN	3.7630	0.0031
	wide, 150: LN vs. RNN	4.9895	0.0012
	wide, 150: NN vs. RNN	11.9631	0.0000
Holm-Bonferroni method for model at wide and 200 ($F_{1.30, 14.34} = 60.0557$, $P < 0.001$)	wide, 200: L vs. C	8.8988	0.0000
	wide, 200: L vs. LN	7.9677	0.0000
	wide, 200: L vs. NN	11.5729	0.0000
	wide, 200: L vs. RNN	6.4623	0.0002
	wide, 200: C vs. LN	6.1320	0.0003
	wide, 200: C vs. NN	10.1088	0.0000
	wide, 200: C vs. RNN	4.6271	0.0022
	wide, 200: LN vs. NN	4.0968	0.0035
	wide, 200: LN vs. RNN	3.4864	0.0051
	wide, 200: NN vs. RNN	10.7583	0.0000
Holm-Bonferroni method for model at wide and 250 ($F_{1.40, 15.43} = 53.0260$, $P < 0.001$)	wide, 250: L vs. C	8.3110	0.0000
	wide, 250: L vs. LN	7.4972	0.0001
	wide, 250: L vs. NN	11.0283	0.0000
	wide, 250: L vs. RNN	6.0647	0.0004
	wide, 250: C vs. LN	5.6775	0.0006
	wide, 250: C vs. NN	9.7577	0.0000
	wide, 250: C vs. RNN	4.3579	0.0034
	wide, 250: LN vs. NN	4.1514	0.0034

		wide, 250: LN vs. RNN	2.3900	0.0359
		wide, 250: NN vs. RNN	9.1325	0.0000
Fig. 5d	Holm-Bonferroni method for phase	narrow vs. square	2.0838	0.0613
		narrow vs. wide	5.0415	0.0008
		square vs. wide	11.1444	0.0000
	Holm-Bonferroni method for time length	50 vs. 100	20.0389	0.0000
		50 vs. 150	20.7478	0.0000
		50 vs. 200	21.5646	0.0000
		50 vs. 250	22.5466	0.0000
		100 vs. 150	21.2697	0.0000
		100 vs. 200	22.0738	0.0000
		100 vs. 250	23.0821	0.0000
		150 vs. 200	22.7381	0.0000
		150 vs. 250	23.8174	0.0000
		200 vs. 250	24.8278	0.0000
	Holm-Bonferroni method for model	L vs. C	17.6430	0.0000
		L vs. LN	6.1188	0.0004
		L vs. NN	12.1332	0.0000
		L vs. RNN	10.0133	0.0000
		C vs. LN	4.0256	0.0021
		C vs. NN	9.7113	0.0000
		C vs. RNN	7.6818	0.0001
		LN vs. NN	5.8433	0.0004
		LN vs. RNN	4.6422	0.0021
		NN vs. RNN	4.6163	0.0021
	Holm-Bonferroni method for condition at 50 ($F_{1.11, 12.21}$ = 21.1788, P < 0.001)	50: narrow vs. square	2.5090	0.0290
		50: narrow vs. wide	5.2306	0.0006
		50: square vs. wide	10.7129	0.0000

Holm-Bonferroni method for condition at 100 ($F_{1.11, 12.25}$ = 21.3673, P < 0.001)	100: narrow vs. square	2.4290	0.0335
	100: narrow vs. wide	5.2061	0.0006
	100: square vs. wide	10.7839	0.0000
Holm-Bonferroni method for condition at 150 ($F_{1.12, 12.29}$ = 21.0753, P < 0.001)	150: narrow vs. square	2.2534	0.0456
	150: narrow vs. wide	5.1321	0.0007
	150: square vs. wide	10.9272	0.0000
Holm-Bonferroni method for condition at 200 ($F_{1.12, 12.31}$ = 20.7208, P < 0.001)	200: narrow vs. square	2.0662	0.0632
	200: narrow vs. wide	5.0420	0.0008
	200: square vs. wide	11.1034	0.0000
Holm-Bonferroni method for condition at 250 ($F_{1.12, 12.30}$ = 20.0662, P < 0.001)	250: narrow vs. square	1.8479	0.0917
	250: narrow vs. wide	4.9091	0.0009
	250: square vs. wide	11.2824	0.0000
Holm-Bonferroni method for time length at narrow ($F_{1.00, 11.04}$ = 137.2361, P < 0.001)	narrow: 50 vs. 100	10.8274	0.0000
	narrow: 50 vs. 150	11.1094	0.0000
	narrow: 50 vs. 200	11.4317	0.0000
	narrow: 50 vs. 250	11.7219	0.0000
	narrow: 100 vs. 150	11.3215	0.0000
	narrow: 100 vs. 200	11.6406	0.0000
	narrow: 100 vs. 250	11.9192	0.0000
	narrow: 150 vs. 200	11.9115	0.0000

	narrow: 150 vs. 250	12.1667	0.0000
	narrow: 200 vs. 250	12.3985	0.0000
Holm-Bonferroni method for time length at square ($F_{1.01, 11.11}$ = 710.4383, P < 0.001)	square : 50 vs. 100	23.7680	0.0000
	square : 50 vs. 150	24.6357	0.0000
	square : 50 vs. 200	25.6542	0.0000
	square : 50 vs. 250	26.7400	0.0000
	square : 100 vs. 150	25.2484	0.0000
	square : 100 vs. 200	26.2404	0.0000
	square : 100 vs. 250	27.2941	0.0000
	square : 150 vs. 200	27.0116	0.0000
	square : 150 vs. 250	28.0154	0.0000
	square : 200 vs. 250	28.8407	0.0000
Holm-Bonferroni method for time length at wide ($F_{1.01, 11.09}$ = 321.0657, P < 0.001)	wide: 50 vs. 100	15.8941	0.0000
	wide: 50 vs. 150	16.4390	0.0000
	wide: 50 vs. 200	17.1495	0.0000
	wide: 50 vs. 250	17.9765	0.0000
	wide: 100 vs. 150	16.8164	0.0000
	wide: 100 vs. 200	17.5392	0.0000
	wide: 100 vs. 250	18.3699	0.0000
	wide: 150 vs. 200	18.1222	0.0000
	wide: 150 vs. 250	18.9492	0.0000
	wide: 200 vs. 250	19.6573	0.0000
Holm-Bonferroni method for condition at L ($F_{1.15, 12.60}$ = 15.8225, P = 0.001)	L: narrow vs. square	1.7755	0.1034
	L: narrow vs. wide	4.4264	0.0020
	L: square vs. wide	9.0532	0.0000
Holm-Bonferroni method for condition at C ($F_{1.13, 12.44}$ = 15.3172, P = 0.002)	C: narrow vs. square	1.9032	0.0835
	C: narrow vs. wide	4.3082	0.0025

	C: square vs. wide	8.6103	0.0000
Holm-Bonferroni method for condition at LN ($F_{1.37, 15.06}$ = 99.2303, P < 0.001)	LN: narrow vs. square	4.4721	0.0009
	LN: narrow vs. wide	11.3309	0.0000
	LN: square vs. wide	15.2963	0.0000
Holm-Bonferroni method for condition at NN ($F_{1.07, 11.73}$ = 15.9297, P = 0.001)	NN: narrow vs. square	1.9656	0.0751
	NN: narrow vs. wide	4.4616	0.0019
	NN: square vs. wide	12.1816	0.0000
Holm-Bonferroni method for condition at RNN ($F_{1.06, 11.63}$ = 14.7834, P = 0.002)	RNN: narrow vs. square	1.6838	0.1203
	RNN: narrow vs. wide	4.2827	0.0026
	RNN: square vs. wide	13.4633	0.0000
Holm-Bonferroni method for model at narrow ($F_{1.13, 12.41}$ = 22.4627, P < 0.001)	narrow: L vs. C	9.5122	0.0000
	narrow: L vs. LN	3.1620	0.0362
	narrow: L vs. NN	7.1086	0.0002
	narrow: L vs. RNN	6.5481	0.0003
	narrow: C vs. LN	2.2647	0.0894
	narrow: C vs. NN	6.3360	0.0004
	narrow: C vs. RNN	5.6591	0.0009
	narrow: LN vs. NN	3.1343	0.0362
	narrow: LN vs. RNN	3.7661	0.0156
	narrow: NN vs. RNN	0.1319	0.8974
Holm-Bonferroni method for model at square	square: L vs. C	10.0611	0.0000
	square: L vs. LN	7.1717	0.0001

($F_{1.25, 13.79}$ = 67.3452,	square: L vs. NN	10.5345	0.0000
P < 0.001)	square: L vs. RNN	8.9136	0.0000
	square: C vs. LN	4.6009	0.0015
	square: C vs. NN	8.7759	0.0000
	square: C vs. RNN	7.1598	0.0001
	square: LN vs. NN	9.6372	0.0000
	square: LN vs. RNN	7.2520	0.0001
	square: NN vs. RNN	4.5721	0.0015
Holm-Bonferroni method	wide: L vs. C	8.2875	0.0000
for model at wide	wide: L vs. LN	7.5267	0.0001
($F_{1.41, 15.52}$ = 52.9709,	wide: L vs. NN	11.1033	0.0000
P < 0.001)	wide: L vs. RNN	6.0480	0.0004
	wide: C vs. LN	5.6620	0.0006
	wide: C vs. NN	9.8002	0.0000
	wide: C vs. RNN	4.3243	0.0036
	wide: LN vs. NN	4.1057	0.0036
	wide: LN vs. RNN	2.4086	0.0347
	wide: NN vs. RNN	8.8879	0.0000
Holm-Bonferroni method	L: 50 vs. 100	17.1596	17.1596
for time length at L	L: 50 vs. 150	17.5602	17.1596
($F_{1.01, 11.06}$ = 350.2917,	L: 50 vs. 200	18.1756	17.1596
P < 0.001)	L: 50 vs. 250	18.7489	17.1596
	L: 100 vs. 150	17.8495	17.1596
	L: 100 vs. 200	18.5378	17.1596
	L: 100 vs. 250	19.1066	17.1596
	L: 150 vs. 200	19.1379	17.1596
	L: 150 vs. 250	19.6196	17.1596
	L: 200 vs. 250	20.0305	17.1596
Holm-Bonferroni method	C: 50 vs. 100	15.4945	0.0000
for time length at C	C: 50 vs. 150	16.1302	0.0000
($F_{1.01, 11.07}$ = 311.5995,	C: 50 vs. 200	16.8854	0.0000
P < 0.001)	C: 50 vs. 250	17.6794	0.0000

	C: 100 vs. 150	16.6311	0.0000
	C: 100 vs. 200	17.4123	0.0000
	C: 100 vs. 250	18.2044	0.0000
	C: 150 vs. 200	18.1002	0.0000
	C: 150 vs. 250	18.8592	0.0000
	C: 200 vs. 250	19.5288	0.0000
Holm-Bonferroni method for time length at LN ($F_{1.05, 11.6} = 2022.8732$, $P < 0.001$)	LN: 50 vs. 100	36.9064	0.0000
	LN: 50 vs. 150	39.6524	0.0000
	LN: 50 vs. 200	42.2624	0.0000
	LN: 50 vs. 250	45.9806	0.0000
	LN: 100 vs. 150	41.4736	0.0000
	LN: 100 vs. 200	43.3542	0.0000
	LN: 100 vs. 250	46.9598	0.0000
	LN: 150 vs. 200	43.4538	0.0000
	LN: 150 vs. 250	47.6464	0.0000
	LN: 200 vs. 250	51.3714	0.0000
Holm-Bonferroni method for time length at NN ($F_{1.01, 11.10} = 405.9222$, $P < 0.001$)	NN: 50 vs. 100	17.6781	0.0000
	NN: 50 vs. 150	18.4041	0.0000
	NN: 50 vs. 200	19.2626	0.0000
	NN: 50 vs. 250	20.2030	0.0000
	NN: 100 vs. 150	18.9244	0.0000
	NN: 100 vs. 200	19.7605	0.0000
	NN: 100 vs. 250	20.6959	0.0000
	NN: 150 vs. 200	20.3560	0.0000
	NN: 150 vs. 250	21.3209	0.0000
	NN: 200 vs. 250	22.1931	0.0000
Holm-Bonferroni method for time length at RNN ($F_{1.02, 11.22} = 480.3615$, $P < 0.001$)	RNN: 50 vs. 100	21.0496	0.0000
	RNN: 50 vs. 150	21.0691	0.0000
	RNN: 50 vs. 200	21.5173	0.0000
	RNN: 50 vs. 250	22.1371	0.0000
	RNN: 100 vs. 150	20.9713	0.0000
	RNN: 100 vs. 200	21.4628	0.0000

	RNN: 100 vs. 250	22.1161	0.0000
	RNN: 150 vs. 200	21.6027	0.0000
	RNN: 150 vs. 250	22.3049	0.0000
	RNN: 200 vs. 250	22.8552	0.0000
Holm-Bonferroni method	50: L vs. C	24.7531	0.0000
for model at 50	50: L vs. LN	10.0645	0.0000
($F_{1.07, 11.78}$ = 107.0474,	50: L vs. NN	13.7400	0.0000
$P < 0.001$)	50: L vs. RNN	10.8060	0.0000
	50: C vs. LN	7.9149	0.0000
	50: C vs. NN	11.4003	0.0000
	50: C vs. RNN	8.6190	0.0000
	50: LN vs. NN	5.2356	0.0006
	50: LN vs. RNN	0.5642	0.5840
	50: NN vs. RNN	8.2598	0.0000
Holm-Bonferroni method	100: L vs. C	22.1803	0.0000
for model at 100	100: L vs. LN	8.1575	0.0000
($F_{1.15, 12.67}$ = 83.3278,	100: L vs. NN	13.0731	0.0000
$P < 0.001$)	100: L vs. RNN	10.3017	0.0000
	100: C vs. LN	5.7441	0.0004
	100: C vs. NN	10.3822	0.0000
	100: C vs. RNN	7.8153	0.0000
	100: LN vs. NN	6.3510	0.0002
	100: LN vs. RNN	4.1993	0.0015
	100: NN vs. RNN	5.4492	0.0004
Holm-Bonferroni method	150: L vs. C	19.1652	0.0000
for model at 150	150: L vs. LN	6.5724	0.0002
($F_{1.23, 13.51}$ = 65.9983,	150: L vs. NN	12.1322	0.0000
$P < 0.001$)	150: L vs. RNN	9.8025	0.0000
	150: C vs. LN	4.2888	0.0015
	150: C vs. NN	9.5687	0.0000
	150: C vs. RNN	7.3676	0.0001
	150: LN vs. NN	6.3494	0.0002

	150: LN vs. RNN	4.8612	0.0015
	150: NN vs. RNN	4.6893	0.0015
Holm-Bonferroni method for model at 200 ($F_{1.28, 14.08}$ = 57.4234, $P < 0.001$)	200: L vs. C	16.6913	0.0000
	200: L vs. LN	5.6043	0.0006
	200: L vs. NN	11.7300	0.0000
	200: L vs. RNN	9.7786	0.0000
	200: C vs. LN	3.5169	0.0048
	200: C vs. NN	9.3093	0.0000
	200: C vs. RNN	7.4368	0.0001
	200: LN vs. NN	5.9683	0.0005
	200: LN vs. RNN	4.8748	0.0015
	200: NN vs. RNN	4.2741	0.0026
Holm-Bonferroni method for model at 250 ($F_{1.30, 14.32}$ = 50.1592, $P < 0.001$)	250: L vs. C	14.5420	0.0000
	250: L vs. LN	4.8692	0.0020
	250: L vs. NN	11.3301	0.0000
	250: L vs. RNN	9.6881	0.0000
	250: C vs. LN	3.0273	0.0115
	250: C vs. NN	9.1962	0.0000
	250: C vs. RNN	7.5870	0.0001
	250: LN vs. NN	5.3777	0.0011
	250: LN vs. RNN	4.6149	0.0022
	250: NN vs. RNN	3.6505	0.0076
Holm-Bonferroni method for model at narrow and 50 ($F_{1.04, 11.46}$ = 36.6509, $P < 0.001$)	narrow, 50: L vs. C	11.1229	0.0000
	narrow, 50: L vs. LN	5.2279	0.0014
	narrow, 50: L vs. NN	7.6252	0.0001
	narrow, 50: L vs. RNN	6.6836	0.0002
	narrow, 50: C vs. LN	4.4752	0.0038
	narrow, 50: C vs. NN	6.9892	0.0002
	narrow, 50: C vs. RNN	5.9768	0.0006
	narrow, 50: LN vs. NN	3.4846	0.0153
	narrow, 50: LN vs. RNN	2.9809	0.0250
	narrow, 50: NN vs. RNN	2.4109	0.0346

Holm-Bonferroni method for model at narrow and 100 ($F_{1.10, 12.08}$ = 30.3442, $P < 0.001$)	narrow, 100: L vs. C	10.2556	0.0000
	narrow, 100: L vs. LN	4.1921	0.0073
	narrow, 100: L vs. NN	7.4700	0.0001
	narrow, 100: L vs. RNN	6.6953	0.0003
	narrow, 100: C vs. LN	3.2709	0.0149
	narrow, 100: C vs. NN	6.7535	0.0003
	narrow, 100: C vs. RNN	5.8610	0.0007
	narrow, 100: LN vs. NN	3.7984	0.0089
	narrow, 100: LN vs. RNN	4.2114	0.0073
	narrow, 100: NN vs. RNN	1.1244	0.2848
Holm-Bonferroni method for model at narrow and 150 ($F_{1.14, 12.55}$ = 24.2769, $P < 0.001$)	narrow, 150: L vs. C	10.1225	0.0000
	narrow, 150: L vs. LN	3.3777	0.0185
	narrow, 150: L vs. NN	7.0922	0.0002
	narrow, 150: L vs. RNN	6.5344	0.0003
	narrow, 150: C vs. LN	2.4218	0.0678
	narrow, 150: C vs. NN	6.3001	0.0004
	narrow, 150: C vs. RNN	5.6281	0.0009
	narrow, 150: LN vs. NN	3.5590	0.0179
	narrow, 150: LN vs. RNN	4.0921	0.0089
	narrow, 150: NN vs. RNN	0.5653	0.5832
Holm-Bonferroni method for model at narrow and 200 ($F_{1.15, 12.62}$ = 20.6968, $P < 0.001$)	narrow, 200: L vs. C	9.7430	0.0000
	narrow, 200: L vs. LN	2.8977	0.0435
	narrow, 200: L vs. NN	6.9696	0.0002
	narrow, 200: L vs. RNN	6.4902	0.0004
	narrow, 200: C vs. LN	1.9833	0.1457
	narrow, 200: C vs. NN	6.1268	0.0005
	narrow, 200: C vs. RNN	5.5471	0.0010
	narrow, 200: LN vs. NN	3.1629	0.0361
	narrow, 200: LN vs. RNN	3.8248	0.0141
	narrow, 200: NN vs. RNN	0.0386	0.9699
	narrow, 250: L vs. C	8.2232	0.0001
	narrow, 250: L vs. LN	2.5742	0.0776

Holm-Bonferroni method for model at narrow and 250 ($F_{1.15, 12.65} = 17.7409$, $P < 0.001$)	narrow, 250: L vs. NN	6.7828	0.0003
	narrow, 250: L vs. RNN	6.3468	0.0004
	narrow, 250: C vs. LN	1.7211	0.2264
	narrow, 250: C vs. NN	5.9928	0.0006
	narrow, 250: C vs. RNN	5.4343	0.0012
	narrow, 250: LN vs. NN	2.7423	0.0766
	narrow, 250: LN vs. RNN	3.4900	0.0253
	narrow, 250: NN vs. RNN	0.5319	0.6054
Holm-Bonferroni method for model at square and 50 ($F_{1.10, 12.11} = 115.5001$, $P < 0.001$)	square, 50: L vs. C	13.3844	0.0000
	square, 50: L vs. LN	11.1825	0.0000
	square, 50: L vs. NN	13.4958	0.0000
	square, 50: L vs. RNN	10.9561	0.0000
	square, 50: C vs. LN	8.5428	0.0000
	square, 50: C vs. NN	11.1463	0.0000
	square, 50: C vs. RNN	8.5898	0.0000
	square, 50: LN vs. NN	7.3405	0.0000
	square, 50: LN vs. RNN	2.2000	0.0501
	square, 50: NN vs. RNN	9.3200	0.0000
Holm-Bonferroni method for model at square and 100 ($F_{1.16, 12.71} = 82.7786$, $P < 0.001$)	square, 100: L vs. C	11.7282	0.0000
	square, 100: L vs. LN	8.9594	0.0000
	square, 100: L vs. NN	11.7822	0.0000
	square, 100: L vs. RNN	9.5745	0.0000
	square, 100: C vs. LN	6.1237	0.0001
	square, 100: C vs. NN	9.4636	0.0000
	square, 100: C vs. RNN	7.3036	0.0001
	square, 100: LN vs. NN	9.5154	0.0000
	square, 100: LN vs. RNN	6.6116	0.0001
	square, 100: NN vs. RNN	5.8972	0.0001
Holm-Bonferroni method for model at square and 150 ($F_{1.23, 13.48} = 67.1723$,	square, 150: L vs. C	10.5000	0.0000
	square, 150: L vs. LN	7.4456	0.0001
	square, 150: L vs. NN	10.7120	0.0000
	square, 150: L vs. RNN	8.8141	0.0000

$P < 0.001$)	square, 150: C vs. LN	4.6777	0.0012
	square, 150: C vs. NN	8.6456	0.0000
	square, 150: C vs. RNN	6.7874	0.0001
	square, 150: LN vs. NN	9.6586	0.0000
	square, 150: LN vs. RNN	7.4401	0.0001
	square, 150: NN vs. RNN	4.7752	0.0012
Holm-Bonferroni method for model at square and 200 ($F_{1.28, 14.08} = 60.9600$, $P < 0.001$)	square, 200: L vs. C	9.4822	0.0000
	square, 200: L vs. LN	6.5788	0.0001
	square, 200: L vs. NN	10.0023	0.0000
	square, 200: L vs. RNN	8.4942	0.0000
	square, 200: C vs. LN	4.0232	0.0029
	square, 200: C vs. NN	8.3342	0.0000
	square, 200: C vs. RNN	6.8383	0.0001
	square, 200: LN vs. NN	9.7605	0.0000
	square, 200: LN vs. RNN	7.4711	0.0001
	square, 200: NN vs. RNN	4.2227	0.0029
Holm-Bonferroni method for model at square and 250 ($F_{1.34, 14.75} = 57.2070$, $P < 0.001$)	square, 250: L vs. C	8.9008	0.0000
	square, 250: L vs. LN	5.9710	0.0003
	square, 250: L vs. NN	9.5739	0.0000
	square, 250: L vs. RNN	8.3158	0.0000
	square, 250: C vs. LN	3.6574	0.0075
	square, 250: C vs. NN	8.2628	0.0000
	square, 250: C vs. RNN	7.0240	0.0001
	square, 250: LN vs. NN	9.4598	0.0000
	square, 250: LN vs. RNN	7.0631	0.0001
	square, 250: NN vs. RNN	3.3811	0.0075
Holm-Bonferroni method for model at wide and 50 ($F_{1.09, 11.98} = 86.2249$, $P < 0.001$)	wide, 50: L vs. C	9.7044	0.0000
	wide, 50: L vs. LN	9.7098	0.0000
	wide, 50: L vs. NN	11.9279	0.0000
	wide, 50: L vs. RNN	7.5432	0.0000
	wide, 50: C vs. LN	8.6872	0.0000
	wide, 50: C vs. NN	11.0902	0.0000

	wide, 50: C vs. RNN	6.1404	0.0001
	wide, 50: LN vs. NN	1.8254	0.0952
	wide, 50: LN vs. RNN	12.4277	0.0000
	wide, 50: NN vs. RNN	17.9134	0.0000
Holm-Bonferroni method for model at wide and 100 ($F_{1.17, 12.86}$ = 69.9679, $P < 0.001$)	wide, 100: L vs. C	9.9967	0.0000
	wide, 100: L vs. LN	8.6931	0.0000
	wide, 100: L vs. NN	11.8206	0.0000
	wide, 100: L vs. RNN	6.9264	0.0001
	wide, 100: C vs. LN	7.0958	0.0001
	wide, 100: C vs. NN	10.5112	0.0000
	wide, 100: C vs. RNN	5.1541	0.0006
	wide, 100: LN vs. NN	3.6946	0.0035
	wide, 100: LN vs. RNN	5.7468	0.0004
	wide, 100: NN vs. RNN	12.2760	0.0000
Holm-Bonferroni method for model at wide and 150 ($F_{1.29, 14.20}$ = 57.3795, $P < 0.001$)	wide, 150: L vs. C	9.0301	0.0000
	wide, 150: L vs. LN	7.8253	0.0000
	wide, 150: L vs. NN	11.1908	0.0000
	wide, 150: L vs. RNN	6.2939	0.0003
	wide, 150: C vs. LN	5.9572	0.0004
	wide, 150: C vs. NN	9.8143	0.0000
	wide, 150: C vs. RNN	4.4658	0.0029
	wide, 150: LN vs. NN	4.3314	0.0029
	wide, 150: LN vs. RNN	3.1714	0.0089
	wide, 150: NN vs. RNN	10.4745	0.0000
Holm-Bonferroni method for model at wide and 200 ($F_{1.49, 16.35}$ = 46.3133, $P < 0.001$)	wide, 200: L vs. C	7.8147	0.0001
	wide, 200: L vs. LN	7.0839	0.0001
	wide, 200: L vs. NN	10.6152	0.0000
	wide, 200: L vs. RNN	5.6150	0.0008
	wide, 200: C vs. LN	5.0954	0.0014
	wide, 200: C vs. NN	9.2636	0.0000
	wide, 200: C vs. RNN	3.8803	0.0051
	wide, 200: LN vs. NN	4.3070	0.0037

		wide, 200: LN vs. RNN	1.7022	0.1168
		wide, 200: NN vs. RNN	8.1275	0.0000
	Holm-Bonferroni method for model at wide and 250 ($F_{1.74, 19.12}$ = 37.4809, P < 0.001)	wide, 250: L vs. C	6.7517	0.0003
		wide, 250: L vs. LN	6.3059	0.0003
		wide, 250: L vs. NN	9.9582	0.0000
		wide, 250: L vs. RNN	5.0671	0.0018
		wide, 250: C vs. LN	4.3989	0.0043
		wide, 250: C vs. NN	8.9034	0.0000
		wide, 250: C vs. RNN	3.5386	0.0093
		wide, 250: LN vs. NN	4.0785	0.0055
		wide, 250: LN vs. RNN	0.9325	0.3711
		wide, 250: NN vs. RNN	6.5264	0.0003
Fig. 6a	Holm-Bonferroni method for horizontal position at narrow	-	-	-
	Holm-Bonferroni method for horizontal position at square	-	-	-
	Holm-Bonferroni method for horizontal position at wide	-	-	-
